# A tetrasomic inheritance model and likelihood‐based method for mapping quantitative trait loci in autotetraploid species

**DOI:** 10.1111/nph.16413

**Published:** 2020-05-16

**Authors:** Jing Chen, Lindsey Leach, Jixuan Yang, Fengjun Zhang, Qin Tao, Zhenyu Dang, Yue Chen, Zewei Luo

**Affiliations:** ^1^ School of Biosciences The University of Birmingham Birmingham B15 2TT UK; ^2^ Institute of Biostatistics Fudan University Shanghai 200433 China; ^3^ Qinghai Academy of Agricultural and Forestry Sciences Xining Qinghai 810016 China

**Keywords:** autotetraploids, double reduction, mixed chromosome pairing, quantitative trait locus (QTL), *Solanum tuberosum*

## Abstract

- Dissecting the genetic architecture of quantitative traits in autotetraploid species is a methodologically challenging task, but a pivotally important goal for breeding globally important food crops, including potato and blueberry, and ornamental species such as rose. Mapping quantitative trait loci (QTLs) is now a routine practice in diploid species but is far less advanced in autotetraploids, largely due to a lack of analytical methods that account for the complexities of tetrasomic inheritance.
- We present a novel likelihood‐based method for QTL mapping in outbred segregating populations of autotetraploid species. The method accounts properly for sophisticated features of gene segregation and recombination in an autotetraploid meiosis. It may model and analyse molecular marker data with or without allele dosage information, such as that from microarray or sequencing experiments.
- The method developed outperforms existing bivalent‐based methods, which may fail to model and analyse the full spectrum of experimental data, in the statistical power of QTL detection, and accuracy of QTL location, as demonstrated by an intensive simulation study and analysis of data sets collected from a segregating population of potato (*Solanum tuberosum*).
- The study enables QTL mapping analysis to be conducted in autotetraploid species under a rigorous tetrasomic inheritance model.

## Introduction

Most agronomic traits targeted in plant or animal breeding programmes are quantitative or complex traits, including yield and quality traits, and resistance to various biotic and abiotic stresses. Phenotypic variation of these traits is under polygenic control, and to a significant extent is also influenced by environmental factors. By using abundantly distributed genomic DNA polymorphisms, this variation can be mapped onto specific chromosomal regions known as quantitative trait loci (QTLs). Models and methods for QTL mapping have been well established in diploid species. However, the corresponding methods are far less advanced in polyploid species, particularly for autopolyploids, even though this group encompasses evolutionarily and economically important plants and aquaculture animals, including potato (*Solanum tuberosum*, the world's third most important food crop), leek, and horticultural crops such as blueberry and rose. This is largely attributed to the complexities in gene segregation and recombination under polysomic inheritance.

In an autotetraploid genome, such as cultivated potato, the four homologous chromosomes may pair in three possible ways and may show random or preferential bivalent formation in different species or genotypes (Bourke *et al.*, 2017). Alternatively, the four chromosomes may form a quadrivalent, which may lead to the phenomenon of double reduction, where identical alleles carried on the sister chromatids enter into the same gamete, causing systematic allelic segregation distortion. This may occur with a frequency of up to 25% in autotetraploids, but it never occurs in a diploid or allopolyploid meiosis (Luo *et al.*, 2006). We have also shown that recombination frequency between a pair of loci can be as high as 75% under a tetrasomic model, compared with 50% in diploids (Luo *et al.*, 2006). These complexities highlight the substantial differences in the patterns of gene segregation and recombination in autopolyploids compared with diploid species. Additional complexities include a high level of heterozygosity stemming from the outbreeding nature of autotetraploids and a much wider spectrum of gene segregation compared with diploids (Bingham, 1980).

Much research has focused on developing theory and methods for QTL analysis in autotetraploids. Methods have been proposed for genetic linkage analysis and QTL mapping in experimental populations of autopolyploid species (Hackett *et al.*, 2001, 2014, 2017; Cao *et al.*, 2005; Xu *et al.*, 2014), and widely practised in QTL mapping analyses in polyploid species (Massa *et al.*, 2015; Da Silva *et al.*, 2017; Massa *et al.*, 2018; Mengist *et al.*, 2018; Bourke *et al.*, 2019; da Silva Pereira *et al.*, 2019). However, these studies have been based on various assumptions that have substantially avoided some complexities of the analyses, but in doing so have ignored some essential features of autotetrasomic inheritance and practical data analysis. Specifically, these refer to different patterns of gene segregation and recombination due to different pairings of homologous chromosomes during meiosis of autotetraploids. Strictly speaking, none of the existing methods in the literature has thoroughly incorporated these into the development of a method for QTL analyses in autotetraploid species as detailed in the following. To fill this theoretical and methodological gap in the field of quantitative genetics, we developed a novel likelihood‐based method for mapping of QTLs in outbred segregating populations of autotetraploid species.

## Description

In general, there are two key components involved in the development of methods for mapping QTLs (Sen & Churchill, 2001), as detailed in the following. The first component is development of a quantitative genetic model, which links the QTL genes to their phenotypic effects on the trait. The second key step is tetrasomic linkage analysis involving a tested QTL and its surrounding genetic markers.

### An outbred autotetraploid mapping population and data notation

The mapping population is created from crossing two autotetraploid parents, P_1_ and P_2_, and presents the first generation of segregation and recombination of genes carried by the two parental individuals. We consider a linkage map of *m* molecular marker loci, *M*_1_, *M*_2_, …, *M_m_*, at each of which there could be up to eight different alleles segregating within the full‐sib mapping population. Let *r_j_* (j=1,2,…,m-1) be the recombination frequency in the *j*th marker interval flanked by markers *M_j_* and *M_j_*_+1_, and *α_j_* (j=1,2,…,m) be the coefficient of double reduction at the *j*th marker. The parents together with *n* offspring individuals are scored at the marker loci. Let $o_{i}={\left(o_{i,j}\right)}_{j=1,\ldots ,m}$ be a vector of marker phenotype for the *i*th offspring individual at the *m* marker loci. Similarly, $p_{1}={\left(p_{1,j}\right)}_{j=1,\ldots ,m}$ or $p_{2}={\left(p_{2,j}\right)}_{j=1,\ldots m}$ is the marker phenotype for P_1_ or P_2_ at the *m* marker loci. $o_{i,j}$, $p_{1,j}$, and $p_{2,j}$ are given by 1 × 8 vectors for the *j*th marker locus, where 1 (or 0) indicates the presence (or absence) of a particular allele. The marker data may be in a form with or without allele dosage information. The trait phenotypic data $y_{i}\in Y$ and the offspring marker data $o_{i}\in O$ (i=1,…,n) are modelled through the likelihood function of the model parameters $\Omega =\{\Omega_{1},\Omega_{2},\Omega_{3}\}$, as shown in the following:

(Eqn 1) $$\begin{array}{cc} & L\left(\Omega |=O,Y\right)=\prod_{i=1}^{n}\mathrm{Pr}\{o_{i},y_{i}|\Omega_{1},\Omega_{2},\Omega_{3}\}\\ & =\prod_{i=1}^{n}\mathrm{Pr}\left\{y_{i}|o_{i},\Omega_{1},\Omega_{2},\Omega_{3}\right\}\mathrm{Pr}\left\{o_{i}|\Omega_{1},\Omega_{2},\Omega_{3}\right\}\\ & \propto \prod_{i=1}=\sum_{\frac{z_{i,j}\in o_{i,j}}{z_{i,j+1}\in o_{i,j+1}}}\sum_{k=0}^{4}f(y_{i}|q_{\mathrm{ik}},\Omega_{1})\\ & \mathrm{Pr}\left\{q_{\mathrm{ik}}|z_{i,j}z_{i,j+1},\Omega_{2}\right\}\mathrm{Pr}\left\{z_{i,j}z_{i,j+1}|o_{i},\Omega_{3}\right\} \end{array}$$

The model parameters in the likelihood function are organized as follows:

$$\Omega =\left\{\Omega_{1},\Omega_{2},\Omega_{3}\right\}=\left\{\left(\mu ,\theta_{1},\theta_{2},\theta_{3},\theta_{4},\sigma^{2}\right),\left(r_{j},g_{1,j},g_{2,j},g_{1,j+1},g_{2,j+1},q_{P_{1}},q_{P_{2}}\right),\left(\alpha ,r,g_{1},g_{2}\right)\right\}$$

and will be explained in the following sections on formulation of the probability distributions involved in the aforementioned likelihood function.

### Orthogonal quantitative genetic model in autotetraploids

In Eqn 1, $f\left(y_{i}|q_{\mathrm{ik}}=G_{k},\Omega_{1}\right)=\exp\left[-{\left(y_{i}-G_{k}\right)}^{2}/2\sigma^{2}\right]/{\left(2\pi \sigma^{2}\right)}^{1/2}$, with $\Omega_{1}=\left(\mu ,\theta_{1},\theta_{2},\theta_{3},\theta_{4},\sigma^{2}\right)$. The genotypic value of the *k*th genotype ($Q_{k}q_{\left(4-k\right)}$) at a putative biallelic QTL to be mapped is modelled through the orthogonal model $G_{k}=\mu +w_{k1}\theta_{1}+w_{k2}\theta_{2}+w_{k3}\theta_{3}+w_{k4}\theta_{4}$, for k=0,1,...,4, representing the number of trait‐phenotype‐increasing alleles. Here, *µ* and *σ* ^2^ are the population mean and residual variance, $\theta_{i}$ (i=1,…,4) are the monogenic, digenic, trigenic, and quadrigenic genetic effects of the QTL, and $w_{\mathrm{kj}}$ (k=0,1,…,4;j=1,…,4) are the corresponding orthogonal contrast scales for the genetic effects of genotype *k* for the *j*th contrast (j=1,2,...,4). The rationale, statistical properties, and parameter estimation of the orthogonal quantitative genetic model are detailed in our recent work (Chen *et al.*, 2018).

### Tetrasomic linkage analysis for quantitative trait locus mapping in autotetraploids

We previously worked out the probability distribution of 136 possible two‐locus gamete genotypes in the offspring from crossing two autotetraploid parental individuals (Luo *et al.*, 2004). By incorporating a biallelic QTL into this two‐locus linkage analysis, we have worked out the three‐locus gamete genotype distribution, $\rho_{q\wedge m}$, of an autotetraploid individual under a quadrivalent pairing model (Table 1), which is fully characterized by the double reduction parameter *α* at the marker locus closest to the centromere, and the recombination frequency parameters. This probability distribution involves a total of 2080 (136 × 16 − 6 × 12 − 6 × 4) different gamete genotypes at the QTL and its flanking markers. This number reduces to 64 if the homologous chromosomes undergo bivalent pairing in meiosis, as summarized in Supporting Information Table S1. Accordingly, one can work out $\rho_{q|m}$, the conditional probability of a gamete QTL genotype given the genotype of the flanking markers. By assuming random union of gametes from the two parents, we worked out the joint marker–QTL–marker zygote genotype probability distribution involving a total of 4326 400 (2080 × 2080) or 4096 (64 × 64) possible zygote genotypes under quadrivalent or bivalent pairing.

**Table 1:**
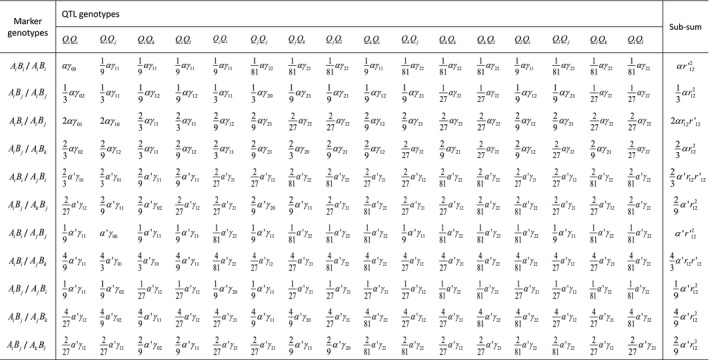
Probability distribution of diploid gamete genotypes at a quantitative trait locus (QTL) and its flanking marker loci from a quadrivalent meiosis of an autotetraploid individual.

where $\gamma_{\mathrm{ij}}=r_{1}^{i}{\left(1-r_{1}\right)}^{2-i}r_{2}^{j}{\left(1-r_{2}\right)}^{2-j}$, *α* is the coefficient of double reduction at locus A and $\alpha^{\prime }=1-\alpha$, *r*_12_ is the recombination frequency between locus A and B, $r_{12}^{\prime }=1-r_{12}$, and *r*_1_ (or *r*_2_) is the recombination frequency between the QTL and its left (or right) flanking marker.

In practice, many offspring genotypes may be identical because there are a smaller number of segregating alleles at the marker loci and can thus be sorted computationally together with their corresponding probabilities. We have developed a computer‐based algorithm and program to handle any number of segregating alleles at the marker loci. These enable calculation of $\mathrm{Pr}\{q_{\mathrm{ik}}|z_{i,j}z_{i,j+1},\Omega_{2}\}$ in Eqn 1, which is the conditional probability of the QTL genotype of individual *i* given its flanking marker genotype and the model parameters $\Omega_{2}=\left(r_{j},g_{1,j},g_{2,j},g_{1,j+1},g_{2,j+1},q_{P_{1}},q_{P_{2}}\right)$. Here, $z_{i,j}z_{i,j+1}$ is the genotype configuration for offspring *i* in the marker interval *j* flanked by markers *M_j_* and *M_j_*_+1_, *r_j_* is the recombination frequency in the *j*th marker interval, *g_i,j_* is the genotype of parent *i* (i=1,2) at the *j*th marker locus, and $q_{P_{i}}$ is the marker–QTL–marker genotype configuration of parent *i* (i.e. with known linkage phase of the marker and QTL alleles). Although the QTL alleles and linkage phase are unknown in practice, we may search all possible configurations over the likelihood function (Eqn 1) and determine the most likely QTL configuration. Moreover, we established $r_{12}=r_{1}+r_{2}-4r_{1}r_{2}/3$ to relate the recombination frequencies in the three‐locus tetrasomic linkage analysis, as detailed in Notes S1.

### Conditional probability of flanking marker genotype

$\mathrm{Pr}\{z_{i,j}z_{i,j+1}|o_{i},\Omega_{3}\}$ in Eqn 1 is the conditional probability of the zygote genotype of individual *i* at the flanking markers *j* and *j* + 1 given all the marker phenotypes on the linkage group and the parental marker genotypes *g*_1_ and *g*_2_ at the marker loci. $\Omega_{3}=\left\{\alpha ,r,g_{1},g_{2}\right\}$, with **α** being a vector of the coefficient of double reduction at the marker loci, **r** is a vector of the recombination frequencies between the adjacent marker loci, and *g*_1_ and *g*_2_ are the parental genotypes at the marker loci. We previously developed a model based on the hidden Markov method to calculate the probability (Leach *et al.*, 2010), as detailed in the present notation in Methods S1.

### Quantitative trait locus interval mapping under different patterns of homologous chromosome pairing

It has been established earlier herein that the conditional probability distribution of QTL genotypes given flanking marker genotypes in an autotetraploid segregating population depends on the pattern of pairing between homologous chromosomes in meiosis. The probability distribution has been developed for quadrivalent (Table 1) or bivalent (Table S1) pairing. These probability distributions may be plugged into a statistically appropriate method for the QTL mapping analysis if one knows which of the two chromosome pairings occurs in the species of interest.

An obvious question arises that chromosome pairing behaviour is usually unknown *a priori*, and the homologous chromosomes may show a mixture of bivalent and quadrivalent pairings, as observed in many autotetraploid species, including potato (Quiros, 1982; Jones *et al.*, 1996; Bradshaw, 2007; Bourke *et al.*, 2015). To tackle the problem, we have first shown in Table 2 that the gamete genotype distribution under mixed chromosomal pairing has an almost identical pattern to that under quadrivalent pairing, except for the difference in value of the coefficient of double reduction. We show that the coefficient of double reduction at a locus under the mixed pairing model (*α*′) can be related to the coefficient in the complete quadrivalent pairing model (*α*) through the simple relationship *α*′ = *λα* (Methods S2), where *λ* is the frequency of quadrivalent chromosome pairing in meiosis. The deviations between the true distribution $g_{i}^{\prime }$ under the mixed pairings (the fifth column in Table 2) and the approximate distribution *ζ_i_* are highlighted in bold. Note that, for a given recombination frequency between the two loci, these deviations will be smaller when *λ* is larger (i.e. when there is a higher frequency of quadrivalent pairing), which will make estimation of gamete probabilities under mixed chromosome pairing more precise. Conversely, when *λ* takes its smallest value of zero (i.e. complete bivalent pairing), the loss of information will be greatest. The Kullback–Leibler divergence from *ζ_i_* to $g_{i}^{\prime }$ is given in the present context by:

(Eqn 2) $$D_{\text{KL}}(g^{\prime }\left||\zeta \right)=-\sum_{i}g_{i}^{\prime }\log\frac{\zeta_{i}}{g_{i}^{\prime }}=-0.301{\left(r-0.585\right)}^{2}+0.103$$

**Table 2:** Probability distribution of diploid gamete genotypes at two linked loci with homologous chromosomes showing quadrivalent, bivalent, or a mixture of the two pairing patterns in an autotetraploid meiosis from an individual with genotype $A_{i}B_{i}/A_{j}B_{j}/A_{k}B_{k}/A_{l}B_{l}$.

Gametes (1≤i,j,k,l≤4)	Double reduction occurred at	Probabilities ($g_{i}$, i=1,…,11)		Gamete genotype probabilities in mixed pairing meiosis	
		Quadrivalent meiosis	Bivalent meiosis	True value ($g_{i}^{\prime }$, i=1,…,11)	Approximation (‍$\zeta_{i}$‍, i=1,…,11‍)
$A_{i}B_{i}/A_{i}B_{i}$	A and B	$\lambda {\left(1-r\right)}^{2}$	—	$\alpha^{\prime }{\left(1-r\right)}^{2}$	$\alpha^{\prime }{\left(1-r\right)}^{2}$
$A_{i}B_{j}/A_{i}B_{j}$	A and B	$\lambda \alpha r^{2}/3$	—	$\alpha^{\prime }r^{2}/3$	$\alpha^{\prime }r^{2}/3$
$A_{i}B_{i}/A_{i}B_{j}$	A	2λαr(1-r)	—	$2\alpha^{\prime }r\left(1-r\right)$	$2\alpha^{\prime }r\left(1-r\right)$
$A_{i}B_{j}/A_{i}B_{k}$	A	$2\lambda \alpha r^{2}/3$	—	$2\alpha^{\prime }r^{2}/3$	$2\alpha^{\prime }r^{2}/3$
$A_{i}B_{i}/A_{j}B_{i}$	B	2λ(1-α)r(1-r)/3	—	$2\left(1-\alpha^{\prime }\right)r\left(1-r\right)/3-2\left(1-\lambda \right)r\left(1-r\right)/3$	$2\left(1-\alpha^{\prime }\right)r\left(1-r\right)/3$
$A_{i}B_{j}/A_{k}B_{j}$	B	$2\lambda \left(1-\alpha \right)r^{2}/9$	—	$2\left(1-\alpha^{\prime }\right)r^{2}/9-2\left(1-\lambda \right)r^{2}/9$	$2\left(1-\alpha^{\prime }\right)r^{2}/9$
$A_{i}B_{i}/A_{j}B_{j}$	—	$\lambda \left(1-\alpha \right){\left(1-r\right)}^{2}$	$\left(1-\lambda \right){\left(1-r\right)}^{2}$	$\left(1-\alpha^{\prime }\right){\left(1-r\right)}^{2}$	$\left(1-\alpha^{\prime }\right){\left(1-r\right)}^{2}$
$A_{i}B_{i}/A_{j}B_{k}$	—	4λ(1-α)r(1-r)/3	2(1-λ)r(1-r)	$4\left(1-\alpha^{\prime }\right)r\left(1-r\right)/3+2\left(1-\lambda \right)r\left(1-r\right)/3$	$4\left(1-\alpha^{\prime }\right)r\left(1-r\right)/3$
$A_{i}B_{j}/A_{j}B_{i}$	—	$\lambda \left(1-\alpha \right)r^{2}/9$	—	$\left(1-\alpha^{\prime }\right)r^{2}/9-\left(1-\lambda \right)r^{2}/9$	$\left(1-\alpha^{\prime }\right)r^{2}/9$
$A_{i}B_{j}/A_{j}B_{k}$	—	$4\lambda \left(1-\alpha \right)r^{2}/9$	—	$4\left(1-\alpha^{\prime }\right)r^{2}/9-4\left(1-\lambda \right)r^{2}/9$	$4\left(1-\alpha^{\prime }\right)r^{2}/9$
$A_{i}B_{j}/A_{k}B_{l}$	—	$2\lambda \left(1-\alpha \right)r^{2}/9$	$\left(1-\lambda \right)r^{2}$	$2\left(1-\alpha^{\prime }\right)r^{2}/9+7\left(1-\lambda \right)r^{2}/9$	$2\left(1-\alpha^{\prime }\right)r^{2}/9$

The coefficient of double reduction in a population undergoing complete quadrivalent (or mixed bivalent and quadrivalent) chromosome pairing is given by *α* (or *α*′), where α′=λα and *λ* is the proportion of quadrivalent pairing in a mixture of quadrivalent and bivalent pairings. Dashes denote genotypes incompatible with bivalent pairing. The terms in bold are those stemming from the mixed homologous chromosome pairings.

Note that if 0.00 < *r* < 0.05, *D*_KL_(*g*′||*ζ*) will vary between 0 and 0.017, reflecting that very little information will be lost by approximating $g_{i}^{\prime }$ with *ζ_i_*. In particular, note from Table 2 that the proportion of gametes with genotypes involving double reduction is the same for the true distribution and the approximate distribution with mixed chromosome pairing (i.e. $\zeta_{1}+\zeta_{2}+\zeta_{3}+\zeta_{4}=g_{1}^{\prime }+g_{2}^{\prime }+g_{3}^{\prime }+g_{4}^{\prime }$). The methods we have previously developed may therefore be used to estimate the average coefficient of double reduction in autotetraploids undergoing a mixture of quadrivalent and bivalent pairings in meiosis (Luo *et al.*, 2000, 2004). These results rationalize use of the QTL mapping method developed in the present study under the quadrivalent pairing model in the case where homologous chromosomes actually undergo a mixture of quadrivalent and bivalent pairing. This will be tested through an intensive simulation study in the following.

### Model parameter estimation

We work out the maximum likelihood estimates (MLEs) of the QTL genotype means as defined in the above, ${\hat{G}}_{k}$ and ${\hat{\sigma }}^{2}$, through the EM algorithm (Dempster *et al.*, 1977) and, in turn, the MLEs of the model parameters $\Omega_{1}=\left(\mu ,\theta_{1},\theta_{2},\theta_{3},\theta_{4},\sigma^{2}\right)$ through iteratively calculating the following two steps.

The E step calculates the probability of the *i*th individual having the *k*th QTL genotype in the *j*th marker interval given its phenotype and marker data as given in the following:

(Eqn 3) $$\omega_{ijq_{k}}=\text{Pr}\left\{q_{\mathrm{ik}}|y_{i},o_{i},\Omega_{1},\Omega_{2},\Omega_{3}\right\}=\sum_{\begin{array}{c} \;\;\;\;\;\;\;\;z_{i,j}\in o_{i,j}\\ z_{i,j+1}\in o_{i,j+1} \end{array}}\frac{f\left(y_{i}|q_{\mathrm{ik}},\Omega_{1}\right)\mathrm{Pr}\left\{q_{\mathrm{ik}}|z_{i,j}z_{i,j+1},\Omega_{2}\right\}\mathrm{Pr}\left\{z_{i,j}z_{i,j+1}|o_{i},\Omega_{3}\right\}}{\sum_{k=0}^{4}f\left(y_{i}|q_{\mathrm{ik}},\Omega_{1}\right)\mathrm{Pr}\left\{q_{\mathrm{ik}}|z_{i,j}z_{i,j+1},\Omega_{2}\right\}}$$

Derivation of this equation is detailed in Methods S3.

The M step then updates the estimates of the genetic parameters from:

(Eqn 4) $$G_{k}^{\prime }=\frac{\sum_{i=1}^{n}\omega_{iq_{k}}y_{i}}{\sum_{i=1}^{n}\omega_{iq_{k}}}$$

(Eqn 5) $$\sigma^{\prime 2}=\sum_{i=1}^{n}\sum_{k=0}^{4}\frac{\omega_{iq_{k}}{\left(y_{i}-G_{k}^{\prime }\right)}^{2}}{n}$$

The iterative algorithm is initiated using the sample variance for *σ* ^2^ and by using *K*‐means clustering to derive initial genotypic values for *G_k_*. As the E and M steps are repeated iteratively following Eqns (Eqn 3), (Eqn 4), (Eqn 5), the likelihood function will increase and the estimated parameters will converge to the MLEs, ${\hat{G}}_{k}$ and ${\hat{\sigma }}^{2}$. The genetic effects at the QTL can be solved from ${\hat{G}}_{k}$ using:

(Eqn 6) $$\left[\begin{array}{c} \hat{\mu }\\ {\hat{\theta }}_{1}\\ {\hat{\theta }}_{2}\\ {\hat{\theta }}_{3}\\ {\hat{\theta }}_{4} \end{array}\right]={\left[\begin{array}{ccccc} 1 & w_{41} & w_{42} & w_{43} & w_{44}\\ 1 & w_{31} & w_{32} & w_{33} & w_{34}\\ 1 & w_{21} & w_{22} & w_{23} & w_{24}\\ 1 & w_{11} & w_{12} & w_{13} & w_{14}\\ 1 & w_{01} & w_{02} & w_{03} & w_{04} \end{array}\right]}^{-1}\left[\begin{array}{c} {\hat{G}}_{4}\\ {\hat{G}}_{3}\\ {\hat{G}}_{2}\\ {\hat{G}}_{1}\\ {\hat{G}}_{0} \end{array}\right]$$

Calculation of *w_ij_*, the orthogonal scales for the genetic effects of QTL genotype *i* (i=0,1,...,4) for the *j*th contrast (j=1,2,...,4), depends on the probability distribution of the QTL genotypes, which can be obtained from the E step in Eqn 3 as detailed in our previous work (Chen *et al.*, 2018). It should be noted that some of the QTL genetic effects may not be estimable for some parental QTL genotype configurations. For example, the trigenic and quadrigenic genetic effects will be indeterminable for the parental QTL genotype configuration *QQqq* × *qqqq* because no relevant offspring QTL genotypes would be generated from the parental QTL genotypes.

With the MLEs of the QTL genotype effects, the likelihood ratio statistic for testing the presence of a QTL at a location characterized by *r_j_*_1_, the recombination frequency between the QTL and its left flanking marker, is calculated as:

(Eqn 7) $$\text{LOD}\left(r_{j1}\right)=\log\left[L\left({\hat{G}}_{k},{\hat{\sigma }}^{2}|O,Y\right)/L\left(\tilde{G},{\tilde{\sigma }}^{2}|O,Y\right)\right]$$

where LOD is the logarithm of the odds. $\tilde{G}$ and ${\tilde{\sigma }}^{2}$ are the estimates of genotypic effects and variance under the no QTL model, given by the mean and variance of the trait calculated from all individuals, assuming the phenotypes to be independently identically normally distributed.

Any possible location within each marker interval may be tested for the presence of QTLs to generate a LOD score profile for each chromosome. Permutation of the offspring phenotypic trait values over the corresponding marker data was used to see how the LOD scores distribute under the null hypothesis that there is no QTL present (Churchill & Doerge, 1994).

The foregoing analysis relies on the availability of information of QTLs and marker genotype and linkage phase information, which is unknown in practice. Assuming parent P_1_ has the higher trait value implying a larger number of trait‐increasing alleles *Q*, there are nine possible genotype configurations for P_1_ and P_2_, listed as (1) QQQQ×QQQq, (2) QQQQ×QQqq, (3) QQQQ×Qqqq, (4) QQQq×QQqq, (5) QQQq×Qqqq, (6) QQQq×qqqq, (7) QQqq×Qqqq, (8) QQqq×qqqq, and (9) Qqqq×qqqq. Taking all possible marker–QTL linkage phases into consideration, there will be up to 92 possible combinations of parental QTL genotypes. QTL interval mapping is carried out for each possible parental QTL genotype configuration. The Bayesian information criterion (BIC) (Schwarz, 1978) is calculated at each location tested as follows:

(Eqn 8) $$\text{BIC}=\log_{e}\left(n\right)k-2\log_{e}\left(\hat{L}\right)$$

where $\hat{L}$ is the maximized value of the likelihood function of the model, *n* is the population size, and *k* is the number of parameters estimated by the model (i.e. the population mean, genetic effects and the residual variance). The most likely parental QTL configuration should have the lowest BIC value, and the estimated genotypic value of parent P_1_ must be higher than that of parent P_2_. Note that there are eight parental QTL configurations for which at least one parental genotype does not appear in the offspring, listed as (1) QQQQ×qqqQ, (2) QQQQ×qqQq, (3) QQQQ×qQqq, (4) QQQQ×Qqqq, (5) QQQq×qqqq, (6) QQqQ×qqqq, (7) QqQQ×qqqq, and (8) qQQQ×qqqq. Under these eight models, we expect to obtain an identical likelihood profile with one of the other 84 parental QTL configurations. For example, the same likelihood profile is expected for genotype configuration (1) QQQQ×qqqQ and an alternative configuration, QQQQ×QQQq. However, the genetic effects would differ in sign under these two models, allowing the correct model to be distinguished, given that the monogenic effect must be positive by definition (Chen *et al.*, 2018).

## Results

### Simulation data analysis

A series of simulation data sets were generated under simulation models I and II, which simulated different pairings of homologous chromosomes during meiosis, marker densities on simulated chromosomes, and particularly generated marker data either with (model I) or without (model II) allele dosage information, as detailed in Notes S2. These simulation data sets were analysed using the QvMethod (the method developed in the present study for modelling quadrivalent chromosome pairing) and/or the BvMethod (the method formulated in the study for modelling bivalent pairing). The methods showed 100% power to detect QTL with a trait heritability of 5% or 10% in a first‐generation segregating (S_1_) population of 300 individuals from crossing two autotetraploid parental lines differing by one, two, or three trait‐increasing alleles under the corresponding chromosome pairing model. Prediction accuracy of the parental QTL genotype configuration depends heavily on the heritability of the simulated QTL and improves when the difference in the number of the trait‐increasing alleles between the parental QTL genotypes increases. Incorrect prediction of QTL genotype configuration may lead to bias in the estimation of higher order genic (e.g. tri or quadrigenic) effects at the QTL (Tables S2, S3). The inferred location of QTLs was within 10 cM of the simulated position in > 70% cases when the heritability was 10%, whereas an expected reduction in accuracy to 44–67% was observed when heritability was only 5%. Similar results were obtained for simulation model II where marker allele dosage information is unknown, as explained in Notes S2 (Tables S4, S5). These results indicate that both BvMethod and QvMethod enable powerful detection of QTLs in outbred autotetraploid populations of a reasonable size and can accurately estimate QTL location and genetic effect parameters when the pairing behaviour of homologous chromosomes is known *a priori* to involve exclusively bivalent or exclusively quadrivalent pairing during meiosis.

To explore the robustness and appropriateness of implementing BvMethod or QvMethod to analyse the data generated under mixed quadrivalent and bivalent chromosomal pairing, we conducted a series of simulations involving 20 biallelic markers on a 100 cM chromosome, with parental QTL genotype QQQq×Qqqq and a trait heritability of 10%. Each population was generated from a given frequency *λ* of quadrivalent chromosome pairing, ranging from *λ* = 0 (complete bivalent pairing) to *λ* = 1 (complete quadrivalent pairing). It must be noted that when BvMethod is implemented with the simulation data generated with *λ* > 0, a proportion of offspring marker genotypes will be incompatible (i.e. not expected to be observed) under the assumption of bivalent chromosome pairing. Incompatibilities may arise in two different ways. First, due to the occurrence of double reduction; for example, a one‐locus gamete with genotype *A*_1_ *A*_1_ must have resulted from double reduction in a parent with genotype *A*_1_ *A*_2_ *A*_3_ *A*_4_. Second, the offspring haplotype must come from no more than two parental chromosomes if bivalent pairing occurs during meiosis. For example, a three‐locus gamete genotype *A*_1_ *B*_1_ *C*_1_/*A*_2_ *B*_3_ *C*_4_ is incompatible with bivalent pairing.

Table 3 summarizes the means and SEs of the MLEs of genetic effects at the QTL, and the empirical power across varying frequencies of quadrivalent pairing from *λ* = 0 to *λ* = 1. Individuals with incompatible genotypes at any of the 20 markers were removed from the data set before analysis with BvMethod. As the frequency of quadrivalent chromosomal pairing increased, estimation of higher order (digenic and trigenic) QTL genetic effects from BvMethod became increasingly biased. Note that the offspring genotypes needed for estimation of the quadrigenic effect (*θ*_4_) are not present under the bivalent chromosomal pairing model. QvMethod estimated the higher order genetic effects much more accurately than BvMethod did, though note that the variance is inherently higher for higher level genetic effects, as detailed in Notes S3. The proportion of correctly predicted parental QTL genotype configurations dropped dramatically using BvMethod, even when the proportion of quadrivalent pairing was low (*λ* = 0.25), with QvMethod correctly predicting parental QTL genotype 68% of the time, compared with only 49% using BvMethod.

**Table 3:** Parameter estimation from BvMethod or QvMethod based on 200 repeated simulations under a mixed chromosome pairing model.

Parameter (=true value)	*λ* = 0.00		*λ* = 0.25		*λ* = 0.50		*λ* = 0.75		*λ* = 1.00	
	BvMethod	QvMethod	BvMethod	QvMethod	BvMethod	QvMethod	BvMethod	QvMethod	BvMethod	QvMethod
*μ* (=10)	10.12 (0.10)	10.14 (0.10)	9.93 (0.10)	9.56 (0.10)	10.15 (0.11)	9.78 (0.13)	10.35 (0.13)	9.39 (0.19)	10.52 (0.13)	10.03 (0.11)
*θ*_1_ (=10)	8.76 (0.17)	8.55 (0.16)	8.69 (0.19)	8.90 (0.17)	7.98 (0.28)	9.06 (0.17)	7.98 (0.29)	9.25 (0.18)	7.99 (0.31)	9.37 (0.15)
*θ*_2_ (=−6)	−4.58 (0.40)	−4.78 (0.40)	−4.59 (0.44)	−4.95 (0.44)	−3.59 (0.53)	−5.04 (0.38)	−3.47 (0.62)	−5.13 (0.35)	−3.19 (0.66)	−5.43 (0.30)
*θ*_3_ (=3)	−6.99 (1.79)	−6.15 (1.84)	−4.76 (1.40)	−0.62 (1.01)	−11.75 (1.75)	1.53 (0.80)	−12.96 (2.02)	2.09 (0.76)	−12.67 (1.98)	1.86 (0.74)
*θ*_4_ (=−1)	—	—	—	−1.18 (2.77)	—	−3.62 (2.24)	—	0.87 (2.46)	–	0.38 (2.25)
$\hat{\sigma }$	21.62 (0.07)	21.64 (0.07)	22.60 (0.07)	22.61 (0.07)	23.63 (0.08)	23.63 (0.07)	24.58 (0.08)	24.69 (0.07)	25.13 (0.09)	25.25 (0.08)
*σ*_true_	21.69		22.67		23.60		24.50		25.35	
Individuals removed	—	—	0.0828 (0.0009)	—	0.1670 (0.0015)	—	0.2515 (0.0017)	—	0.3344 (0.0019)	—
Empirical Power	1.00	1.00	1.00	1.00	1.00	1.00	0.99	1.00	0.98	1.00
*Q*_genotype0_	0.660	0.665	0.490	0.680	0.395	0.675	0.345	0.715	0.260	0.755
*Q*_genotype1_	0.980	0.980	0.965	0.990	0.86	0.975	0.835	0.980	0.815	0.995
Accuracy (cM)	1.66 (0.66)	1.75 (0.73)	3.51 (0.74)	2.75 (0.54)	5.68 (0.98)	2.17 (0.59)	8.395 (1.206)	3.05 (0.65)	10.145 (1.262)	2.56 (0.66)
Proportion in (± 10 cM)	0.845	0.830	0.820	0.860	0.720	0.840	0.600	0.820	0.575	0.855

The simulated parental quantitative trait locus (QTL) genotype was QQQq×Qqqq. The proportion of quadrivalent chromosome pairing is given by *λ*. *Q*_genotype0_ (*Q*_genotype1_) represents the proportion of predicted parental QTL genotype configurations exactly matching (differing by only a single allele) the simulated QTL genotypes. The mean and SEs of parameter estimates are given based on 200 replicate simulations. Individuals with at least one marker genotype that is unexpected from the theoretical genotype distribution under a model of bivalent chromosome pairing are removed from the simulated data sets before analysis with BvMethod. Accuracy (cM) is the distance between the true QTL location and its inferred location. Heritability was equal to 10%.

QvMethod is substantially more accurate than BvMethod in locating the QTLs when the homologous chromosomes show a mixture of both bivalent and quadrivalent pairings. As quadrivalent pairing increases in frequency, the accuracy of BvMethod drops markedly.

For example, at *λ* = 0.50, the QTL detected is 5.68 ± 0.98 cM away from the true QTL location using BvMethod, but this is narrowed down to only 2.17 ± 0.59 cM by using QvMethod. At higher values of *λ*, the improvement in accuracy through use of QvMethod becomes more pronounced. Note also that performance of QvMethod is comparable to that of BvMethod even when the homologous chromosomes undergo pure bivalent pairing (*λ* = 0). A similar pattern of results was obtained in the analyses where individual incompatible genotypes were treated as missing data, as others (Hackett *et al.*, 2014, 2017) have done elsewhere (Tables S6, S7). The degree of improvement in accuracy through use of QvMethod will naturally depend on the particular parental genotype configuration (Tables S6, S7), and hence on how extreme the difference is between the true offspring genotype distribution and the genotype distribution obtained under the assumption of bivalent pairing.

### Experimental data analysis

We demonstrated the utility of QvMethod in real experimental data analysis using single nucleotide polymorphism (SNP) and phenotype data sets collected by Bradshaw *et al.* (2008) and repeatedly analysed by Hackett *et al.* (2014, 2017). The data were collected from 190 offspring individuals generated from a cross between two autotetraploid parental potato lines, ‘Stirling’ and 12601ab1. As described elsewhere (Hackett *et al.*, 2017), this outbred segregating population was scored for 12 quantitative traits. The methods described in Hackett *et al.* (2001, 2014) have recently been engineered into the Windows‐supported software tetraploidsnpmap, which enables genetic linkage map reconstruction and QTL mapping analysis in autotetraploids (Hackett *et al.*, 2017). The software is illustrated by mapping QTLs for the 12 agronomic traits on linkage group V reconstructed with 119 SNP markers, which corresponds to potato (*S. tuberosum*) chromosome V. Individual marker genotypes incompatible with the assumption of bivalent pairing were removed from the data by the authors. To demonstrate the method we have developed (i.e. QvMethod) and enable a direct comparison with the method in TetraploidSNPMap (abbreviated here as H2017), we focused here on QTL mapping analysis with the 12 traits on linkage group V (though we have scanned QTLs for these traits over all 12 potato chromosomes). In the real data analysis, an empirical mapping resolution was defined as the size (in centimorgans) of the chromosomal interval over which the LOD score profile dropped by a value of 2.0 on both sides of the QTL peak.

**Figure 1:**
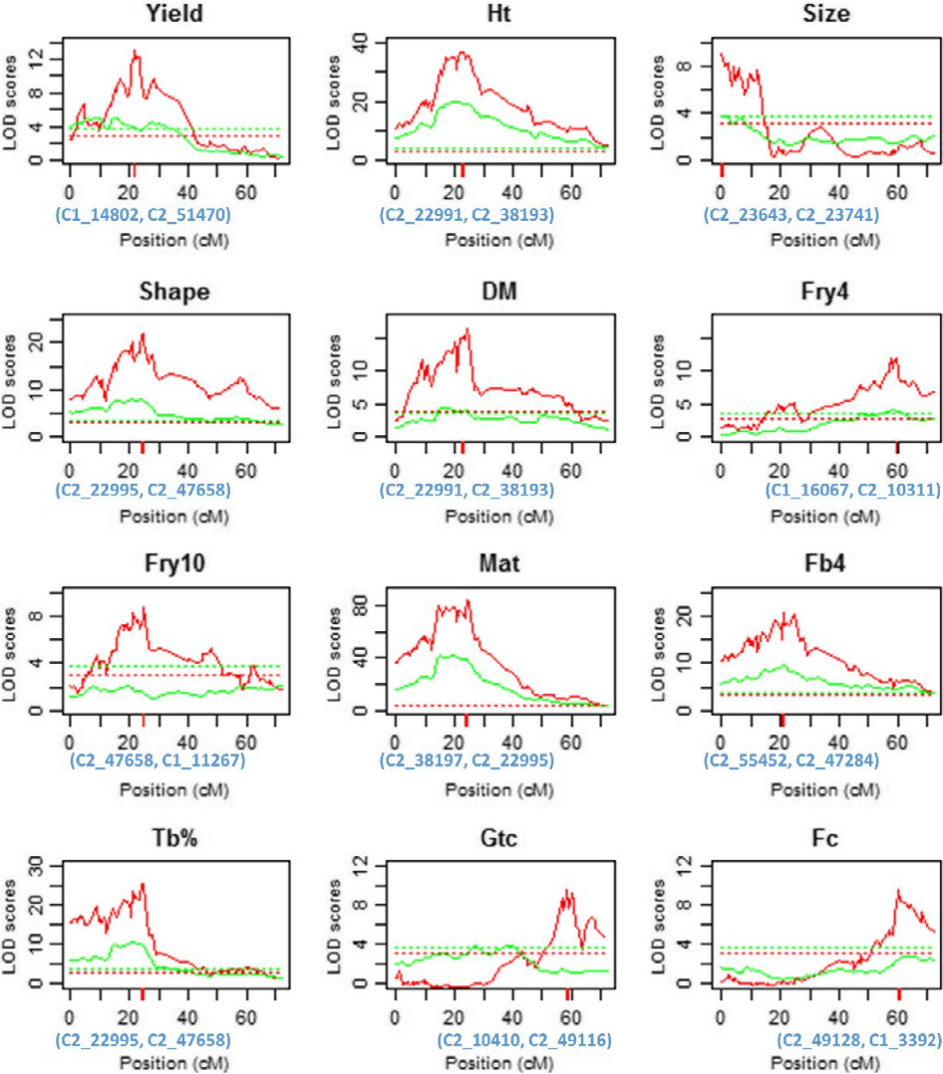
Logarithm of odds (LOD) score profiles of quantitative trait loci (QTLs) mapped on potato chromosome V for 12 agronomic traits of autotetraploid potato (*Solanum tuberosum*). The LOD score profiles were derived from QvMethod (red lines) and H2017 (green lines). The dotted lines represent the corresponding significance thresholds from 100 permutation tests. The red bars indicate the most likely chromosomal locations of the QTLs detected by QvMethod, and listed on the chromosomal *x*‐axes are the markers closest to the detected QTLs. The 12 traits are yield (fresh tuber weight in kilograms per plot), Ht (canopy height), size (tuber), shape (tuber), DM (dry matter), Fry4 and Fry10 (frying colour at 4°C and 10°C), Mat (maturity), Fb4 (foliage blight), Tb% (tuber blight), Gtc (*Globodera pallida* cyst counts), and Fc (flower colour). H2017 represents the method developed in Hackett *et al.* (2017).

Fig. 1 illustrates the LOD score profiles of QTL detection for QvMethod (red lines) and H2017 (green lines) for the 12 potato agronomic traits. The significance thresholds (dotted lines) were calculated from 100 permutation tests of the LOD score test statistic for the corresponding method. QvMethod clearly shows a markedly higher statistical power for detecting the QTLs than the bivalent‐pairing‐based H2017 method does. In particular, QvMethod detected QTLs for four of the 12 traits (tuber size, Fry10, *Globodera* cyst counts, and flower colour) that were missed by the H2017 method (Fig. 1; Table 2). Table 4 shows that QvMethod confers a substantially higher QTL mapping resolution than the H2017 method does. Table 2 also shows that the QTLs detected by QvMethod usually explain a larger proportion of genetic variance of the trait (Vg%) compared with the QTLs detected by H2017. These observations suggest that H2017, a method strictly based on a bivalent chromosomal pairing model, may fail to detect QTLs that have a small genetic effect on a quantitative trait. For a given Vg%, the estimated proportion of additive genetic variance of the QTLs detected, $\hat{\rho }$, indicates that the QTLs detected by both methods contribute largely additive genetic variation to the trait phenotype variation. The notable exception is tuber yield, which may reflect the particularly complex genetic architecture of this trait, both in potato (Schonhals *et al.*, 2017) and other crops (Shi *et al.*, 2009). Note that three of the four QTLs that were missed by H2017 (Fry10, Gtc, Fc) have a relatively small additive genetic variance, reflecting the fact that H2017 only models additive QTL genetic effects.

**Table 4:** Empirical mapping resolution and estimates of genetic effects of quantitative trait loci (QTL) on chromosome V for 12 traits in potato (*Solanum tuberosum*).

Trait	Resolution (cM)		Vg (%)		QvMethod parameter estimates							
	QvMethod	H2017	QvMethod	H2017	Parental QTL	$\hat{\mu }$	${\hat{\theta }}_{1}$	${\hat{\theta }}_{2}$	${\hat{\theta }}_{3}$	${\hat{\theta }}_{4}$	$\hat{\sigma }$	$\hat{\rho }$ (%)
Yield	4	38	9.04	7.88	QqQQ×QqQq	7.98	0.16	−1.29	1.29	6.61	1.76	5.52
Ht	2	10	32.08	35.59	QQQq×qqqq	53.00	8.55	11.97	—	—	6.5	95.90
Size	3	ns	8.46	ns	qqQQ×qqQq	4.29	0.26	−0.18	−0.55	1.45	0.76	77.23
Shape	3	23	23.01	14.59	qqqQ×QqqQ	4.26	0.37	0.39	−0.22	−0.51	0.60	77.86
Dm	3	35	14.59	6.82	qqqQ×QqqQ	22.14	0.47	0.65	−1.54	−1.68	1.21	55.00
Fry4	3	37	11.58	6.03	qQqQ×Qqqq	4.08	0.28	−0.21	0.18	0.88	0.66	85.74
Fry10	11	ns	4.30	ns	qQqQ×QqQQ	6.34	0.13	−0.37	0.19	0.04	0.63	31.29
Mat	3	8	57.08	57.91	QQQq×qqqq	4.76	2.27	2.17	—	—	1.01	98.49
Fb4	1	14	19.78	17.89	QQQq×qqqq	4.87	1.77	−0.48	—	—	1.81	99.91
Tb%	3	13	26.48	20.70	qqqQ×qqqq	56.94	27.88	27.95	—	—	23.78	98.50
Gtc	4	ns	12.44	ns	QQqQ×Qqqq	4.26	0.33	1.27	−1.68	−4.96	1.34	22.47
Fc	5	ns	11.75	ns	qqQq×qQqQ	1.44	0.14	0.04	0.81	1.68	0.46	43.10

The empirical QTL mapping resolution is defined as the chromosomal region in centimorgans over which the logarithm of odds score drops by a value of 2.0 on each side of the QTL peak. Vg (%) is the proportion of genetic variance explained by the QTL detected. In the predicted parental QTL genotype, *Q* (or *q*) indicates the trait increasing (or decreasing) allele. $\hat{\mu }$, ${\hat{\theta }}_{1}$, ${\hat{\theta }}_{2}$, ${\hat{\theta }}_{3}$, ${\hat{\theta }}_{4}$, and $\hat{\sigma }$ are the estimated population mean, monogenic, digenic, trigenic, quadrigenic effects, and residual error for the QTL detected based on the orthogonal contrast quantitative genetic model. $\hat{\rho }$ is the estimated proportion of genetic variance explained by additive QTL genetic effects. The 12 traits are yield (fresh tuber weight in kilograms per plot), Ht (canopy height), size (tuber), shape (tuber), DM (dry matter), Fry4 and Fry 10 (frying colour at 4°C and 10°C), Mat (maturity), Fb4 (foliage blight), Tb% (tuber blight), Gtc (*Globodera pallida* cyst counts), and Fc (flower colour). Dashes denote indeterminable parameters, because the relevant offspring genotypes are not observable for the given predicted parental QTL genotypes. ns denotes nonsignificant QTL. H2017 represents the method described in Hackett *et al.* (2017).

### Significance of chromosomal pairing patterns in quantitative trait locus mapping efficiency

The previously mentioned simulation study and real data analysis demonstrated that QvMethod outperforms other methods that assume only bivalent chromosomal pairing in the meiosis of autotetraploids, both in terms of QTL detection power and mapping resolution. This thus urges the essentiality of modelling the complex features of tetrasomic gene segregation and recombination for QTL mapping in autotetraploid species and motivated us to explore the difference in statistical efficiency between the two types of method.

**Figure 2:**
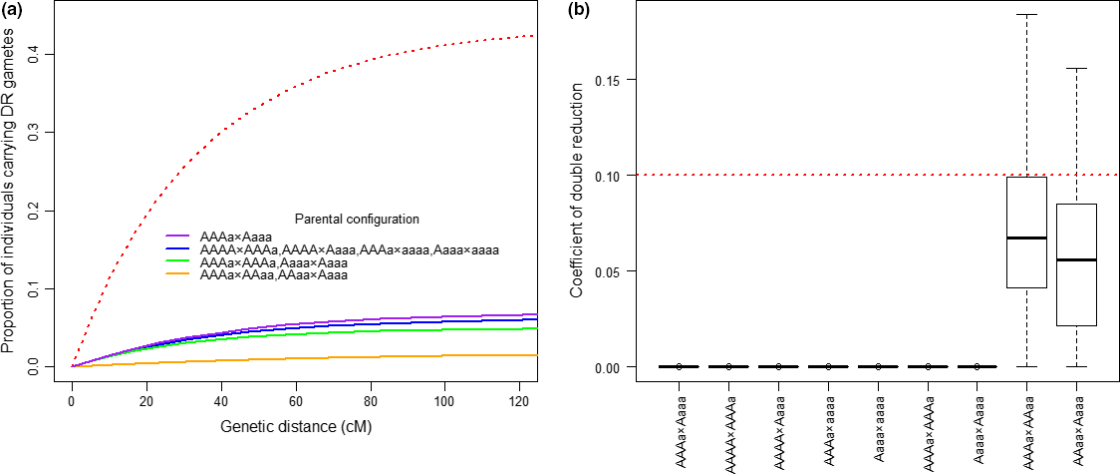
Occurrence and estimation of double reduction at a single locus in an offspring population from crossing two outbred autotetraploids under a quadrivalent pairing model. An offspring population of 200 individuals was simulated for a single locus based on a cross between two outbred parents with nine possible parental quantitative trait locus (QTL) genotype configurations. (a) The expected (red dotted line) and observable (solid lines) proportion of individuals carrying double reduction gametes in the population from crossing different parental QTL genotypes. (b) Distribution of estimates for the coefficient of double reduction in the population from crossing nine possible parental QTL genotypes (labelled on horizontal axis) when offspring individuals carrying observable double reduction gametes were excluded from the data set before the analysis. The simulated value is indicated by the red dotted line. Each simulation was repeated 100 times.

We explored how the different chromosomal pairings affect the genetic structure of mapping populations. It is clear from Table 2 that the genotypic distribution of the mapping population differs substantially according to the mode of chromosome pairing (bivalent or quadrivalent), not only in the probability of a genotype, but also in the number of possible genotypes. In particular, if homologous chromosomes undergo quadrivalent pairing, there could be a substantial proportion of individuals in the mapping population carrying gametes from double reduction events (hereafter referred to as double reduction gametes) and hence being incompatible with the assumption of bivalent pairing. Fig. 2(a) (red dotted line) shows that the expected proportion of individuals carrying double reduction gametes increases with distance from the centromere and could be as high as 40%, but only a small number of incompatible offspring genotypes are observable. Furthermore, for three of the 12 possible parental genotype configurations, no incompatible offspring genotypes can be observed. A substantial gap therefore exists between the incompatible genotypes that can be observed directly from the marker data (Fig. 2a, coloured solid lines) and the expected number of incompatible genotypes (Fig. 2a, red dotted line). We also investigated the influence of excluding individuals with observable incompatible marker data on estimation of the level of double reduction, based on 100 repeated simulations with 200 offspring individuals from crossing two autotetraploid parents. The distribution of double reduction estimates is far below the simulated value for each of nine possible parental genotype configurations when the incompatible data are removed (Fig. 2b). This shows how excluding only a small (i.e. the observable) fraction of incompatible individuals is very sensitive in weakening the signal of double reduction.

These observations largely, if not fully, explain why the development of QvMethod in this study outperforms the bivalent‐pairing‐based methods when all or some of the homologous chromosomes undergo quadrivalent pairing during meiosis, and indicate that the bivalent‐pairing‐based methods, strictly speaking, cannot truly model and analyse the full spectrum of data from real QTL mapping experiments in autotetraploid species.

## Discussion

Mapping of QTLs is a key initial step towards understanding the molecular mechanisms underpinning quantitative genetic variation. It has taken decades from the conception of an idea (Sax, 1923) to maturation of the methods (Lander & Botstein, 1989; Sen & Churchill, 2001) in diploid species, and QTL mapping is now a routine genetic analysis in almost all evolutionarily and economically important diploid species. However, linkage analysis with autotetraploids has remained a historical challenge since the work of prominent geneticists such as J. B. S. Haldane, K. Mather, and R. A. Fisher (Haldane, 1930; Mather, 1936; Fisher, 1947). Although a breakthrough on this topic was made in our previous work (Luo *et al*, 2004, 2006; Leach *et al.*, 2010), linkage analysis involving QTLs under a tetrasomic inheritance model, the theoretical basis for mapping QTLs, has remained a theoretical bottleneck and a methodological gap in the field of quantitative genetic analysis with autotetraploid species.

Here, we have presented a theoretical and methodological breakthrough that enables QTL mapping to be carried out in autotetraploid species on the basis of a rigorous tetrasomic inheritance model. The methods account properly for the key features of gene segregation and recombination when homologous chromosomes undergo bivalent and quadrivalent pairing, as well as a mixture of the two modes of chromosome pairing in the meiosis of autotetraploid species. Hackett *et al.* (2001, 2014, 2017) pioneered the development of statistical methods for QTL mapping in autotetraploids that assume exclusively bivalent chromosomal pairing, and have been widely cited in the literature. This assumption may substantially simplify the statistical analyses so as to enable the methodology development, but it comes at the price of sacrificing the key features of tetrasomic inheritance in autotetraploid species.

Quadrivalent chromosomal pairing has been observed in various autotetraploid plants, with a frequency varying from up to 10% in kiwi (Wu *et al.*, 2014), 20–30% in potato (Bourke *et al.*, 2015), and up to 36% in *Pennisetum orientale* (Deniz & Dogru, 2006). In these cases, bivalent‐based methods cannot deal with the full spectrum of the data because there will be a large proportion of observed data that is incompatible with the expected distribution under the bivalent pairing assumption. In practice, it has been proposed to exclude the incompatible data from QTL mapping analysis when using bivalent‐based methods (Hackett *et al.*, 2014, 2017; da Silva Pereira *et al.*, 2019). We have shown that the chance to directly observe the incompatible data is very low or even impossible, because a substantial proportion of individuals carry gametes from double reduction events that are undetectable in the mapping population and therefore cannot be excluded from the data. This creates a serious problem, whereby the genetic structure of the mapping population deviates significantly from that expected under bivalent chromosome pairing, even when the observable incompatible data are removed. Our development of QvMethod accounts for quadrivalent chromosome pairing and therefore outperforms the bivalent‐based methods both in terms of statistical power of QTL detection and accuracy of locating QTLs, as demonstrated through simulation study and analysis with real data from a segregating population of autotetraploid potato (*S. tuberosum*). Recently, Bourke *et al.* (2019) explored factors affecting QTL analysis in polyploids by combining the computer software ‘tetraorigin’, which was designed by Zheng *et al.* (2016) to predict the parental origin of marker alleles in autotetraploid populations, and a simplified additive genetic model into QTL mapping analysis. However, the method described in Bourke *et al.* (2019) lacks an essential component; that is, genetic linkage analysis between QTLs and surrounding markers for QTL mapping in an outbred autotetraploid segregating population, as we have presented here.

The QTL mapping method presented here is based on a biallelic quantitative genetic model detailed in our recent work (Chen *et al.*, 2018). A full multiple allele quantitative genetic model for a tetraploid individual, such as the one defined in Kempthorne (1957), is statistically intractable because it involves a total of 96 genetic parameters (Hackett *et al.*, 2001). This number increases to 120 in a segregating population from crossing two parental lines divergent at all four alleles of a QTL (8 monogenic, 28 digenic, 48 trigenic, and 36 quadrigenic effects). A simplified version of this multiple allele model has been implemented by either setting linear constraints on the genetic effects, as in Hackett *et al.* (2001, 2014, 2017), or by modelling only some of the genetic effects of a full model (e.g. additive effects), as in Bourke *et al.* (2019). Using these simplified models, estimates of the genetic parameters may be biased and difficult to interpret because many other genetic effects (e.g. interactive effects between QTL alleles) are ignored. To solve the intractability problem and to adequately estimate the genetic parameters in a full model, we proposed an orthogonal contrast‐based model for modelling quantitative genetic effects in various autotetraploid populations, as detailed in Chen *et al.* (2018), and implement it in the present QTL mapping method. The biallelic model focuses on genetic effects of QTL alleles on trait phenotypes and models their increasing or decreasing effects on the traits. It is statistically tractable and completely models all genetic effects (additive and interactive) at a putative QTL. In particular, the model does not imply that there are only two alleles on the molecular level (i.e. sequence variants), but instead the two ‘alleles’ define the increasing or decreasing quantitative genetic effects of QTL alleles on the trait.

In addition to trait phenotype data, molecular marker data present the other essential component in any QTL analysis. New‐generation genomic DNA sequencing techniques enable discovery and generation of a large number of DNA sequence polymorphic markers in a tested genome. Genotyping by sequencing (GBS) is relatively straightforward in diploid species, though serious consideration must be given to several major sources of variation in collecting and processing the sequencing data for accurate identification of allele‐specific sequencing reads (Geijn *et al.*, 2015). GBS in tetraploids is a much more challenging task and involves distinguishing the number of each constituent allele (i.e. the allele dosage) in a heterozygote genotype (e.g. Uitdewilligen *et al.*, 2013). Though methods for diagnosis of allele dosage from DNA sequencing data in tetraploids are beginning to emerge (Margarido & Heckerman, 2015; Gerard *et al.*, 2018), this task remains an incredible challenge and is ripe for further investigation. The QTL methods developed in this study can utilize marker data with or without known allele dosage information. Hackett *et al.* (2014) showed that use of marker dosage allele information may improve the efficiency of QTL mapping analysis. An open question arises as to what extent the biased diagnosis of allele dosage would affect the efficiency of mapping QTLs in autotetraploids.

## Author contributions

ZL conceived of and designed the study. ZL and JC designed the theoretical model and statistical methods. JC and ZL implemented the statistical methods. JC analysed the data with inputs from ZL and LL. Contributions to data collection and preparation of the project were made by JY, FZ, QT, ZD and YC. The paper was written by ZL, JC and LL.

## Supporting information


**Methods S1** Conditional probability of flanking marker genotypes given their surrounding marker data.
**Methods S2** Relationship between the coefficients of double reduction at linked loci under the mixed chromosome pairing model.
**Methods S3** The EM algorithm for maximum likelihood estimation of QTL genetic effects.
**Notes S1** Relationship between recombination frequencies of QTL and flanking markers under quadrivalent and bivalent chromosome pairing models.
**Notes S2** Simulation model and parameters.
**Notes S3** Variance in the estimation of genetic effect parameters.
**Table S1** Probability distribution of diploid gamete genotypes at a QTL and its flanking marker loci from a bivalent meiosis of an autotetraploid individual.
**Table S2** Parameter estimation from BvMethod based on 100 simulations with biallelic SNP markers under a bivalent chromosome pairing model.
**Table S3** Parameter estimation from QvMethod based on 100 simulations with biallelic SNP markers under a quadrivalent chromosome pairing model.
**Table S4** Parameter estimation from BvMethod based on 100 simulations with multi‐allelic markers under a bivalent chromosome pairing model.
**Table S5** Parameter estimation from BvMethod or QvMethod based on 200 repeated simulations under a mixed chromosome pairing model.
**Table S6** Parameter estimation from BvMethod or QvMethod based on 200 repeated simulations under a mixed chromosome pairing model with simulated parental QTL genotypes *QQQq* × *Qqqq*.
**Table S7** Parameter estimation from BvMethod or QvMethod based on 200 repeated simulations under a mixed chromosome pairing model with simulated parental QTL genotypes *QQqq* × *qqqq.*
Please note: Wiley Blackwell are not responsible for the content or functionality of any Supporting Information supplied by the authors. Any queries (other than missing material) should be directed to the *New Phytologist* Central Office.

## Data Availability

The methods, BvMethod and QvMethod, developed in this paper have been built into an R package and can be downloaded together with the data sets analysed in the present study from Figshare with permanent https://doi.org/10.6084/m9.figshare.11298179.

## References

[nph16413-bib-0001] Bingham ET . 1980. Maximizing heterozygosity in autopolyploids. In: Lewis WH , ed. Polyploidy: biological relevance. Boston, MA, USA: Springer, 471–489. 10.1007/978-1-4613-3069-1_24 550838

[nph16413-bib-0002] Bourke PM , Arens P , Voorrips RE , Esselink GD , Koning‐Boucoiran CF , van't Westende WPC , Santos Leonardo T , Wissink P , Zheng C , van Geest G *et al*. 2017. Partial preferential chromosome pairing is genotype dependent in tetraploid rose. The Plant Journal 90: 330–343. 28142191 10.1111/tpj.13496

[nph16413-bib-0003] Bourke PM , Hackett CA , Voorrips RE , Visser RGF , Maliepaard C . 2019. Quantifying the power and precision of QTL analysis in autopolyploids under bivalent and multivalent genetic models. G3: Genes, Genomes, Genetics 9: 2107–2122. 31036677 10.1534/g3.119.400269 PMC6643892

[nph16413-bib-0004] Bourke PM , Voorrips RE , Visser RG , Maliepaard C . 2015. The double‐reduction landscape in tetraploid potato as revealed by a high‐density linkage map. Genetics 201: 853–863. 26377683 10.1534/genetics.115.181008 PMC4649655

[nph16413-bib-0005] Bradshaw JE . 2007. The canon of potato science: 4. Tetrasomic inheritance. Potato Research 50: 219–222.

[nph16413-bib-0006] Bradshaw JE , Hackett CA , Pande B , Waugh R , Bryan B . 2008. QTL mapping of yield, agronomic and quality traits in tetraploid potato (*Solanum tuberosum* subsp. *tuberosum*). Theoretical and Applied Genetics 116: 193–211. 17938877 10.1007/s00122-007-0659-1

[nph16413-bib-0007] Cao D , Craig BA , Doerge RW . 2005. A model selection‐based interval‐mapping method for autopolyploids. Genetics 169: 2371–2382. 15687274 10.1534/genetics.104.035410 PMC1449588

[nph16413-bib-0008] Chen J , Zhang F , Wang L , Leach LJ , Luo ZW . 2018. Orthogonal contrast based models for quantitative genetic analysis in autotetraploid species. New Phytologist 220: 332–346. 10.1111/nph.15284 29987874

[nph16413-bib-0009] Churchill GA , Doerge RW . 1994. Empirical threshold values for quantitative trait mapping. Genetics 138: 963–971. 7851788 10.1093/genetics/138.3.963 PMC1206241

[nph16413-bib-0011] Da Silva WL , Ingram J , Hackett CA , Coombs JJ , Douches D , Bryan GJ , De Jong W , Gray S . 2017. Mapping loci that control tuber and foliar symptoms caused by PVY in autotetraploid potato (*Solanum tuberosum* L.). G3: Genes, Genomes, Genetics 7: 3587–3595. 28903982 10.1534/g3.117.300264 PMC5675608

[nph16413-bib-0012] Dempster AP , Laird NM , Rubin DB . 1977. Maximum likelihood from incomplete data via the EM algorithm. Journal of the Royal Statistical Society: Series B (Methodological) 39: 1–38.

[nph16413-bib-0013] Deniz B , Dogru U . 2006. Meiotic behaviour in natural diploid, tetraploid, and commercial diploid crested wheatgrass. New Zealand Journal of Agricultural Research 49: 405–409.

[nph16413-bib-0014] Fisher RA . 1947. The theory of linkage in polysomic inheritance. Philosophical Transactions of the Royal Society of London. Series B: Biological Sciences 23: 55–87.

[nph16413-bib-0015] Gerard D , Ferrao LFV , Garcia AAF , Stephens M . 2018. Genotyping polyploids from messy sequencing data. Genetics 210: 789–807. 30185430 10.1534/genetics.118.301468 PMC6218231

[nph16413-bib-0016] Hackett CA , Boskamp B , Vogogias A , Preedy KF , Milne I . 2017. TetraploidSNPMap: software for linkage analysis and QTL mapping in autotetraploid populations using SNP dosage data. Journal of Heredity 108: 438–442.

[nph16413-bib-0017] Hackett CA , Bradshaw JE , Bryan GJ . 2014. QTL mapping in autotetraploids using SNP dosage information. Theoretical and Applied Genetics 127: 1885–1904. 24981609 10.1007/s00122-014-2347-2 PMC4145212

[nph16413-bib-0018] Hackett CA , Bradshaw JE , McNicol JW . 2001. Interval mapping of quantitative trait loci in autotetraploid species. Genetics 159: 1819–1832. 11779817 10.1093/genetics/159.4.1819 PMC1461889

[nph16413-bib-0019] Haldane JBS . 1930. Theoretical genetics of autopolyploids. Journal of Genetics 22: 359–372.

[nph16413-bib-0020] Jones GH , Khazanehdari KA , Ford‐Lloyd BV . 1996. Meiosis in the leek (*Allium porrum* L.) revisited. II. Metaphase I observations. Heredity 76: 186–191.

[nph16413-bib-0021] Kempthorne O . 1957. An introduction to genetic statistics. New York, NY, USA: John Wiley & Sons.

[nph16413-bib-0022] Lander ES , Botstein D . 1989. Mapping mendelian factors underlying quantitative traits using RFLP linkage maps. Genetics 121: 185–199. 2563713 10.1093/genetics/121.1.185 PMC1203601

[nph16413-bib-0023] Leach LJ , Wang L , Kearsey MJ , Luo ZW . 2010. Multilocus tetrasomic linkage analysis using hidden Markov chain model. Proceedings of the National Academy of Sciences, USA 107: 4270–4274. 10.1073/pnas.0908477107 PMC2840161 20142473

[nph16413-bib-0024] Luo ZW , Hackett CA , Bradshaw JE , McNicol JW , Milbourne D . 2000. Predicting parental genotypes and gene segregation for tetrasomic inheritance. Theoretical and Applied Genetics 100: 1067–1073.

[nph16413-bib-0025] Luo ZW , Zhang RM , Kearsey MJ . 2004. Theoretical basis for genetic linkage analysis in autotetraploid species. Proceedings of the National Academy of Sciences, USA 101: 7040–7045. 10.1073/pnas.0304482101 PMC406462 15100415

[nph16413-bib-0026] Luo ZW , Zhang Z , Leach L , Zhang RM , Bradshaw JE , Kearsey MJ . 2006. Constructing genetic linkage maps under a tetrasomic model. Genetics 172: 2634–2645. 10.1534/genetics.105.052449 PMC1456397 16415363

[nph16413-bib-0027] Margarido GRA , Heckerman D . 2015. ConPADE: genome assembly ploidy estimation from next‐generation sequencing data. PLoS Computational Biology 11: e1004229. 25880203 10.1371/journal.pcbi.1004229 PMC4400156

[nph16413-bib-0028] Massa AN , Manrique‐Carpintero NC , Coombs JJ , Haynes KG , Bethke PC , Brandt TL , Gupta SK , Yencho GC , Novy RG , Douches DS . 2018. Linkage analysis and QTL mapping in a tetraploid russet mapping population of potato. BMC Genetics 19: e87. 30241465 10.1186/s12863-018-0672-1 PMC6150958

[nph16413-bib-0029] Massa AN , Manrique‐Carpintero NC , Coombs JJ , Zarka DG , Boone AE , Kirk WW , Hackett CA , Bryan GJ , Douches DS . 2015. Genetic linkage mapping of economically important traits in cultivated tetraploid potato (*Solanum tuberosum* L.). G3: Genes, Genomes, Genetics 5: 2357–2364. 26374597 10.1534/g3.115.019646 PMC4632055

[nph16413-bib-0030] Mather K . 1936. Segregation and linkage in autotetraploids. Journal of Genetics 32: 287–314.

[nph16413-bib-0031] Mengist MF , Alves S , Griffin D , Creedon J , McLaughlin MJ , Jones PW , Milbourne D . 2018. Genetic mapping of quantitative trait loci for tuber‐cadmium and zinc concentration in potato reveals associations with maturity and both overlapping and independent components of genetic control. Theoretical and Applied Genetics 131: 929–945. 29307117 10.1007/s00122-017-3048-4

[nph16413-bib-0032] Quiros CF . 1982. Tetrasomic segregation for multiple alleles in alfalfa. Genetics 101: 117–127. 17246077 10.1093/genetics/101.1.117 PMC1201845

[nph16413-bib-0033] Sax K . 1923. The association of size difference with seed‐coat pattern and pigmentation in *PHASEOLUS VULGARIS* . Genetics 8: 552–560. 17246026 10.1093/genetics/8.6.552 PMC1200765

[nph16413-bib-0034] Schonhals EM , Ding J , Ritter E , Paulo MJ , Cara N , Tacke E , Hofferbert HR , Lubeck J , Strahwald J , Gebhardt C . 2017. Physical mapping of QTL for tuber yield, starch content and starch yield in tetraploid potato (*Solanum tuberosum* L.) by means of genome wide genotyping by sequencing and the 8.3 K SolCAP SNP array. BMC Genomics 18: e642. 28830357 10.1186/s12864-017-3979-9 PMC5567664

[nph16413-bib-0035] Schwarz G . 1978. Estimating the dimension of a model. Annals of Statistics 6: 461–464.

[nph16413-bib-0036] Sen S , Churchill GA . 2001. A statistical framework for quantitative trait mapping. Genetics 159: 371–87. 11560912 10.1093/genetics/159.1.371 PMC1461799

[nph16413-bib-0037] Shi JQ , Li RY , Qiu D , Jiang CC , Long Y , Morgan C , Bancroft I , Zhao JY , Meng JL . 2009. Unraveling the complex trait of crop yield with quantitative trait loci mapping in *Brassica napus* . Genetics 182: 851–861. 19414564 10.1534/genetics.109.101642 PMC2710164

[nph16413-bib-0010] da Silva Pereira G , Gemenet DC , Mollinari M , Olukolu BA , Wood JC . 2019. Multiple QTL mapping in autopolyploids: a random‐effect model approach with application in a hexaploid sweetpotato full‐sib population. *bioRxiv* . 10.1101/622951 PMC7337090 32371382

[nph16413-bib-0038] Uitdewilligen JGAM , Wolters AMA , D'hoop BB , Borm TJA , Visser RGF , van Eck HJ . 2013. A next‐generation sequencing method for genotyping‐by‐sequencing of highly heterozygous autotetraploid potato. PLoS ONE 8: e62355. 26509671 10.1371/journal.pone.0141940 PMC4624895

[nph16413-bib-0039] van de Geijn B , McVicker G , Gilad Y , Pritchard JK . 2015. Wasp: allele‐specific software for robust molecular quantitative trait locus discovery. Nature Methods 12: 1061–1063. 26366987 10.1038/nmeth.3582 PMC4626402

[nph16413-bib-0040] Wu JH , Datson PM , Manako KI , Murray BG . 2014. Meiotic chromosome pairing behaviour of natural tetraploids and induced autotetraploids of *Actinidia chinensis* . Theoretical and Applied Genetics 127: 549–557. 24306317 10.1007/s00122-013-2238-y

[nph16413-bib-0041] Xu F , Lyu Y , Tong C , Wu W , Zhu X , Yin D , Yan Q , Zhang J , Pang X , Tobias CM *et al*. 2014. A statistical model for QTL mapping in polysomic autotetraploids underlying double reduction. Briefings in Bioinformatics 15: 1044–1056. 24177379 10.1093/bib/bbt073

[nph16413-bib-0042] Zheng C , Voorrips RE , Jansen J , Hackett CA , Ho J , Bink MC . 2016. Probablistic multilocus haplotype reconstruction in outcrossing tetraploids. Genetics 203: 119–131. 26920758 10.1534/genetics.115.185579 PMC4858767

