# Supplementary material for: A tetrasomic inheritance model and likelihood‐based method for mapping quantitative trait loci in autotetraploid species
Source: New Phytol. 2020 May 16;230(1):387–98. doi: 10.1111/nph.16413 (PMC7984458; doi:10.1111/nph.16413)
Supplement: Supplementary file 1 — Methods S1 Conditional probability of flanking marker genotypes given their surrounding marker data. Methods S2 Relationship between the coefficients of double reduction at linked loci under the mixed chromosome pairing model. Methods S3 The EM algorithm for maximum likelihood estimation of QTL genetic effects. Notes S1 Relationship between recombination frequencies of QTL and flanking markers under quadrivalent and bivalent chromosome pairing models. Notes S2 Simulation model and parameters. Notes S3 Variance in the estimation of genetic effect parameters. Table S1 Probability distribution of diploid gamete genotypes at a QTL and its flanking marker loci from a bivalent meiosis of an autotetraploid individual. Table S2 Parameter estimation from BvMethod based on 100 simulations with biallelic SNP markers under a bivalent chromosome pairing model. Table S3 Parameter estimation from QvMethod based on 100 simulations with biallelic SNP markers under a quadrivalent chromosome pairing model. Table S4 Parameter estimation from BvMethod based on 100 simulations with multi‐allelic markers under a bivalent chromosome pairing model. Table S5 Parameter estimation from BvMethod or QvMethod based on 200 repeated simulations under a mixed chromosome pairing model. Table S6 Parameter estimation from BvMethod or QvMethod based on 200 repeated simulations under a mixed chromosome pairing model with simulated parental QTL genotypes QQQq × Qqqq. Table S7 Parameter estimation from BvMethod or QvMethod based on 200 repeated simulations under a mixed chromosome pairing model with simulated parental QTL genotypes QQqq × qqqq. Please note: Wiley Blackwell are not responsible for the content or functionality of any Supporting Information supplied by the authors. Any queries (other than missing material) should be directed to the New Phytologist Central Office. [file NPH-230-387-s001.pdf]

***New Phytologist* Supporting Information**

Article title: A Tetrasomic Inheritance Model and Likelihood-based Method for Mapping Quantitative Trait Loci in Autotetraploid Species

Authors: Jing Chen, Lindsey Leach, Jixuan Yang, Fengjun Zhang, Tao Qin, Zhenyu Dang, Yue Chen and Zewei Luo: 20 December 2019

The following Supporting Information is available for this article:

3 supporting methods,

3 supporting notes

7 supporting tables

SUPPORTING METHODS

Supporting Method S1. Conditional probability of flanking marker genotypes given their surrounding marker data

$\Pr\left\{z_{i,j}z_{i,j+1}\middle|o_i,\Omega_3\right\}$ in equation (1) is the conditional probability of the zygote genotype of individual *i* at the flanking markers *j* and *j*+1 given all the marker phenotypes on the linkage group and the parental marker genotypes g_1 and g_2 at the marker loci. It may be formulated as:

$$\begin{aligned}
&\Pr\left\{z_{i,j}z_{i,j+1}\middle|o_i,\Omega_3\right\}=\Pr\left\{z_{a(i,j)}z_{a(i,j+1)}/z_{b(i,j)}z_{b(i,j+1)}\middle|o_i,\Omega_3\right\}\\
&=\frac{\Pr\left\{z_{a(i,j)}z_{a(i,j+1)}/z_{b(i,j)}z_{b(i,j+1)}\middle|\Omega_3\right\}\Pr\left\{o_{i,1}\ldots o_{i,m}\middle|z_{a(i,j)}z_{a(i,j+1)}/z_{b(i,j)}z_{b(i,j+1)},\Omega_3\right\}}{\sum\limits_{\substack{z_{a(i,j)}/z_{b(i,j)}\in o_{i,j}\\ z_{a(i,j+1)}/z_{b(i,j+1)}\in o_{i,j+1}}}\Pr\left\{z_{a(i,j)}z_{a(i,j+1)}/z_{b(i,j)}z_{b(i,j+1)}\middle|\Omega_3\right\}\Pr\left\{o_{i,1}\ldots o_{i,m}\middle|z_{a(i,j)}z_{a(i,j+1)}/z_{b(i,j)}z_{b(i,j+1)},\Omega_3\right\}}\\
&=\frac{\Pr\left\{z_{a(i,j)}z_{a(i,j+1)}/z_{b(i,j)}z_{b(i,j+1)}\middle|\Omega_3\right\}\Pr\left\{o_{i,1}\ldots o_{i,j}\middle|z_{a(i,j)}/z_{b(i,j)},\Omega_3\right\}\Pr\left\{o_{i,j+1}\ldots o_{i,m}\middle|z_{a(i,j+1)}/z_{b(i,j+1)},\Omega_3\right\}}{\sum\limits_{\substack{z_{a(i,j)}/z_{b(i,j)}\in o_{i,j}\\ z_{a(i,j+1)}/z_{b(i,j+1)}\in o_{i,j+1}}}\Pr\left\{z_{a(i,j)}z_{a(i,j+1)}/z_{b(i,j)}z_{b(i,j+1)}\middle|\Omega_3\right\}\Pr\left\{o_{i,1}\ldots o_{i,j}\middle|z_{a(i,j)}/z_{b(i,j)},\Omega_3\right\}\Pr\left\{o_{i,j+1}\ldots o_{i,m}\middle|z_{a(i,j+1)}/z_{b(i,j+1)},\Omega_3\right\}}
\end{aligned}\qquad\text{(MS1)}$$

where $\Omega_3=\left\{\boldsymbol{\alpha},\boldsymbol{r},g_1,g_2\right\}$, in which $\boldsymbol{\alpha}$ is a vector of the coefficient of double reduction at the marker loci, $\boldsymbol{r}$ is a vector of the recombination frequencies between the adjacent marker loci, and g_1 and g_2 are the parental genotypes at the marker loci. These parameters can be estimated from the marker data by using the methods we previously developed **(Luo et al. 2004; Luo et al. 2000)**. The above simplification is possible because of the Markov property of the genotype distribution at linked loci, *i.e.* given the marker genotype M_j, the genotype distributions of markers to the left of M_j will be independent of the marker genotype of M_{j+1}. Therefore, given the genotypes of markers M_j, and M_{j+1}, the phenotypes of markers $M_1,\ldots,M_j$ would be independent of the phenotypes of markers $M_{j+1},\ldots,M_m$. By assuming random union of gametes from the two parents, the first term in equation (MS1) can be calculated as a product of the probabilities of the two-locus gamete genotypes as given below:

$$\Pr\left\{z_{a(i,j)}z_{a(i,j+1)}/z_{b(i,j)}z_{b(i,j+1)}\middle|\Omega_3\right\}=\Pr\left\{z_{a(i,j)}z_{a(i,j+1)}\middle|\boldsymbol{\alpha},\boldsymbol{r},g_1\right\}\Pr\left\{z_{b(i,j)}z_{b(i,j+1)}\middle|\boldsymbol{\alpha},\boldsymbol{r},g_2\right\}$$

$\Pr\left\{z_{a(i,j)}z_{a(i,j+1)}\middle|\alpha_j,r_j,g_1\right\}$ and $\Pr\left\{z_{b(i,j)}z_{b(i,j+1)}\middle|\alpha_j,r_j,g_2\right\}$ are the gamete genotype probabilities given parental genotypes g_1 and g_2 respectively, and the relevant elements are listed in **Table 4**. The second ($\Pr\left\{o_1\ldots o_j\middle|z_{a(i,j)}/z_{b(i,j)},\Omega_3\right\}$) and third ($\Pr\left\{o_{j+1}\ldots o_m\middle|z_{a(i,j+1)}/z_{b(i,j+1)},\Omega_3\right\}$) terms in

equation (MS1) are calculated using the Hidden Markov chain model, as shown in equations (4) and (5) of our previous work **(Leach et al. 2010)**.

Supporting Method S2. Relationship between the coefficients of double reduction at linked loci under the mixed chromosome pairing model

Consider a locus at which the coefficient of double reduction is given by α under complete quadrivalent pairing. The gamete genotype at the locus is given by $\Pr\{A_iA_i \mid \alpha\} = \alpha/4$ and $\Pr\{A_iA_j \mid \alpha\} = (1-\alpha)/6$ with $1 \le i \ne j \le 4$, which can be found elsewhere **(Luo et al. 2000)**. If, however, quadrivalent pairing accounts for a proportion λ of total pairings, with the remaining proportion 1 – λ involving bivalent pairing, then the gamete genotype distribution at a single locus showing mixed chromosome pairing is given by:

$$\begin{cases} \Pr\{A_iA_i \mid \alpha,\lambda\} = \alpha\lambda/4 & 1 \le i \le 4 \\ \Pr\{A_iA_j \mid \alpha,\lambda\} = (1-\lambda\alpha)/6 & 1 \le i \ne j \le 4 \end{cases} \qquad \text{(MS2)}$$

This indicates that the coefficient of double reduction at the locus under a mixed pairing meiosis model can be related to that in the complete quadrivalent pairing model through the relationship $\alpha' = \lambda\alpha$.

Table 3 shows the gamete genotype distribution at two loci *A* and *B* in an autotetraploid meiosis in which homologous chromosomes undergoing quadrivalent, bivalent or a mixture of the two chromosomal pairings. Under complete quadrivalent pairing, we previously established that α, the coefficient of double reduction at locus A, is related to β, the coefficient of double reduction at locus B, through $\beta = \left[\alpha(3-4r)^2 + 2r(3-2r)\right]/9$ **(Luo et al. 2004)**. From the probabilities of gamete genotypes involving double reduction at locus B, *i.e.* g_1, g_2, g_5 and g_6 in the third column of **Table 3** we can work out the coefficient of double reduction at this locus to be:

$$\beta' = g_1 + g_2 + g_5 + g_6 = \lambda\left[\alpha(3-4r)^2 + 2r(3-2r)\right]/9 = \lambda\beta$$

Together with the relationship between β and β' , this indicates the invariance property of the influence of mixed chromosome pairing on the double reduction parameters at the linked loci, therefore rationalizing use of a quadrivalent pairing model to approximate the gamete genotype distribution under mixed chromosome pairing in the tetrasomic linkage analysis.

Supporting Method S3. The EM algorithm for maximum likelihood estimation of QTL genetic effects

Consider a full-sib outbred segregating population from crossing two autotetraploid individuals. Given the trait phenotypic values, $y_i \in Y$, and the observed offspring marker data, $o_i \in O$ $(i = 1, ..., n)$ of the *n* offspring individuals, the likelihood of the model parameters Ω can be formulated as shown in equation (1) of **Models and Methods** as

$$L(\Omega|O,Y) = \prod_{i=1}^{n} \Pr\{o_i, y_i|\Omega_1, \Omega_2, \Omega_3\} = \prod_{i=1}^{n} \Pr\{y_i|o_i, \Omega_1, \Omega_2, \Omega_3\} \Pr\{o_i|\Omega_1, \Omega_2, \Omega_3\}$$
$$\propto \prod_{i=1}^{n} \Pr\{y_i|o_i, \Omega_1, \Omega_2, \Omega_3\} = \sum_{\substack{z_{i,j} \in o_{i,j} \\ z_{i,j+1} \in o_{i,j+1}}} \sum_{k=o}^{4} f(y_i|q_{ik}, \Omega_1) \Pr\{q_{ik}|z_{i,j}z_{i,j+1}, \Omega_2\} \Pr\{z_{i,j}z_{i,j+1}|o_i, \Omega_3\}$$

where $\Omega = \{\Omega_1, \Omega_2, \Omega_3\} = \{(\mu, \theta_1, \theta_2, \theta_3, \theta_4, \sigma^2), (r_j, g_{1,j}, g_{2,j}, g_{1,j+1}, g_{2,j+1}, q_{P_1}, q_{P_2}), (\boldsymbol{\alpha}, \boldsymbol{r}, g_1, g_2)\}$, each of which has been explained in context.

Here, we focused on working out the maximum likelihood estimates of the QTL genotype means G_k ($k = 0, 1, \ldots, 4$) corresponding to the number of increasing effect QTL alleles, and the residual variance σ^2. The genetic effects at the QTL, $\theta_1, \theta_2, \theta_3$ and θ_4 can be derived directly from G_k as formulated in our previous work **(Chen et al. 2018)**. First, the E-step of the EM algorithm calculates the probability of the *i*th individual having the *k*th QTL genotype at a test location within the *j*th marker interval, conditional on the individual's trait phenotype and the flanking marker genotype given an initial set of the model parameters as:

$$\omega_{iq_k} = \Pr\{q_k|y_i, o_i, \Omega\}$$
$$= \sum_{\substack{z_{i,j} \in o_{i,j} \\ z_{i,j+1} \in o_{i,j+1}}} \Pr\{q_{ik}|y_i, z_{i,j}z_{i,j+1}, \Omega\} \Pr\{z_{i,j}z_{i,j+1}|o_i, \Omega\}$$

According to Bayes theorem, $\Pr\{q_{ik}|y_i, z_{i,j}z_{i,j+1}, \Omega\}$ can be calculated as:

$$\Pr\{q_{ik}|y_i, z_{i,j}z_{i,j+1}, \Omega\} = \frac{f(y_i|q_{ik}, z_{i,j}z_{i,j+1}, \Omega) \Pr\{q_{ik}|z_{i,j}z_{i,j+1}, \Omega\}}{\sum_{k=0}^{4} f(y_i|q_{ik}, z_{i,j}z_{i,j+1}, \Omega) \Pr\{q_{ik}|z_{i,j}z_{i,j+1}, \Omega\}}$$

which can be substituted into the above equation for ω_{iq_k} to give:

$$\omega_{iq_k} = \sum_{\substack{z_{i,j}\in o_{i,j} \\ z_{i,j+1}\in o_{i,j+1}}} \frac{f\left(y_i \middle| q_{ik}, z_{i,j} z_{i,j+1}, \Omega\right) \Pr\left\{q_{ik} \middle| z_{i,j} z_{i,j+1}, \Omega\right\} \Pr\left\{z_{i,j} z_{i,j+1} \middle| o_i, \Omega\right\}}{\sum_{k=0}^{4} f\left(y_i \middle| q_{ik}, z_{i,j} z_{i,j+1}, \Omega\right) \Pr\left\{q_{ik} \middle| z_{i,j} z_{i,j+1}, \Omega\right\}}$$

Removing the irrelevant parameters in the above probabilities, this equation can be simplified to give equation (3) in **Models and Methods** as follows:

$$\omega_{iq_k} = \sum_{\substack{z_{i,j}\in o_{i,j} \\ z_{i,j+1}\in o_{i,j+1}}} \frac{f\left(y_i \middle| q_{ik}, \Omega_1\right) \Pr\left\{q_{ik} \middle| z_{i,j} z_{i,j+1}, \Omega_2\right\} \Pr\left\{z_{i,j} z_{i,j+1} \middle| o_i, \Omega_3\right\}}{\sum_{k=0}^{4} f\left(y_i \middle| q_{ik}, \Omega_2\right) \Pr\left\{q_{ik} \middle| z_{i,j} z_{i,j+1}, \Omega_2\right\}}$$

References to Supporting Methods

Chen J, Zhang F, Wang L, Leach LJ, Luo ZW. 2018. Orthogonal contrast based models for quantitative genetic analysis in autotetraploid species. *New Phytol*. **220**: 332-346.

Luo ZW, Hackett CA, Bradshaw JE, McNicol JW, Milbourne D. 2000. Predicting parental genotypes and gene segregation for tetrasomic inheritance. *Theor Appl Genet* **100:** 1067-1073.

Luo ZW, Zhang RM, Kearsey MJ. 2004. Theoretical basis for genetic linkage analysis in autotetraploid species. *Proc Natl Acad Sci USA* **101:** 7040-7045.

Leach LJ, Wang L, Kearsey MJ, Luo ZW. 2010. Multilocus tetrasomic linkage analysis using hidden Markov chain model. *Proc Natl Acad Sci USA* **107:** 4270-4274.

SUPPORTING TEXT

Supporting Note S1. Relationship between recombination frequencies of QTL and flanking markers under quadrivalent and bivalent chromosome pairing models

We consider three loci A, B and C linked on an autotetraploid chromosome. Let A_i, B_i and C_i be the alleles at the three loci, with *i* indexing the four homologous chromosomes of an autotetraploid individual. r_1, r_2 and r_{12} are the recombination frequencies between loci A and B, loci B and C, and loci A and C, respectively. Assuming a Poisson distribution of crossovers along the chromosome and an absence of recombination interference, we develop the relationship between the recombination frequencies. This relationship depends on two alternative modes of homologous chromosome pairing during meiosis, which we discuss separately below.

Quadrivalent Pairing

Note S1 Fig. 1 illustrates quadrivalent pairing of 8 duplicated chromatids and possible recombination occurring across the three loci. The probability of recombination between loci A and C, *i.e.* r_{12}, is equivalent to the probability that A_1 and C_1 will not be on the same chromosome strand after recombination. The recombination may occur between alleles A_1 and B_1 on strand 1. For example, A_1 on strand 1 is connected to B_2 on strand 3 as shown in **Text S1 Fig. 1**.

Note S1 Fig. 1. Recombination events in a three locus linkage model for autotetraploid species under quadrivalent chromosome pairing.

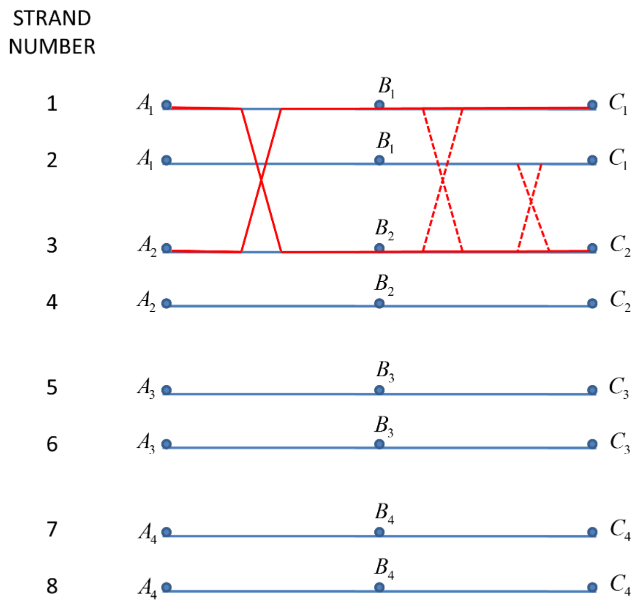

Eight blue lines represent the duplicated homologous chromosomes in autotetraploids, with markers *A*, *B* and *C* locating along the chromosome. The red lines indicate recombination events occurring between non-sister chromatids.

In this case, two of the possible six recombination events for strand 1 in the second interval *BC* would restore the connection between A_1 and C_1 on strand 1. The probability that A_1 on strand 1 will not link to C_1 is therefore $r_1(1-r_2/3)$. Alternatively, if there is no recombination between alleles A_1 and B_1 on strand 1, then recombination between alleles A_1 and C_1 will happen only if B_1 recombines to C_2, C_3 or C_4 on the remaining six strands, with a probability of $(1-r_1)r_2$. The probability that A_1 on strand 1 will not link to C_1 after recombination is therefore equal to $r_1(1-r_2/3)+(1-r_1)r_2$. The relationship between r_{12}, r_1 and r_2 is therefore

$$r_{12} = r_1(1-r_2/3)+(1-r_1)r_2 = r_1 + r_2 - 4r_1r_2/3 \quad \text{(S1)}$$

r_2 can be solved for a given r_{12} and r_1 as

$$r_2 = \frac{r_{12}-r_1}{1-4r_1/3} \quad \text{(S2)}$$

Bivalent pairing

Note S1 Fig. 2 shows that duplicated homologous chromosomes randomly pair as bivalents. There are three possible pairings among the four homologous chromosomes and recombination occurs between paired chromosomes, as in diploids. An example illustrated in this figure shows chromosome 1 pairing with chromosome 2, and the remaining chromosomes 3 and 4 pairing together. It is well known that $r_{12} = r_1 + r_2 - 2r_1r_2$ holds for paired chromosomes, which will be obtained by averaging over the three possible pairings of the homologous chromosomes in autotetraploids.

SUPPORTING Note S1 Fig 2. Chromosome pairing in a bivalent meiosis of an autotetraploid species.

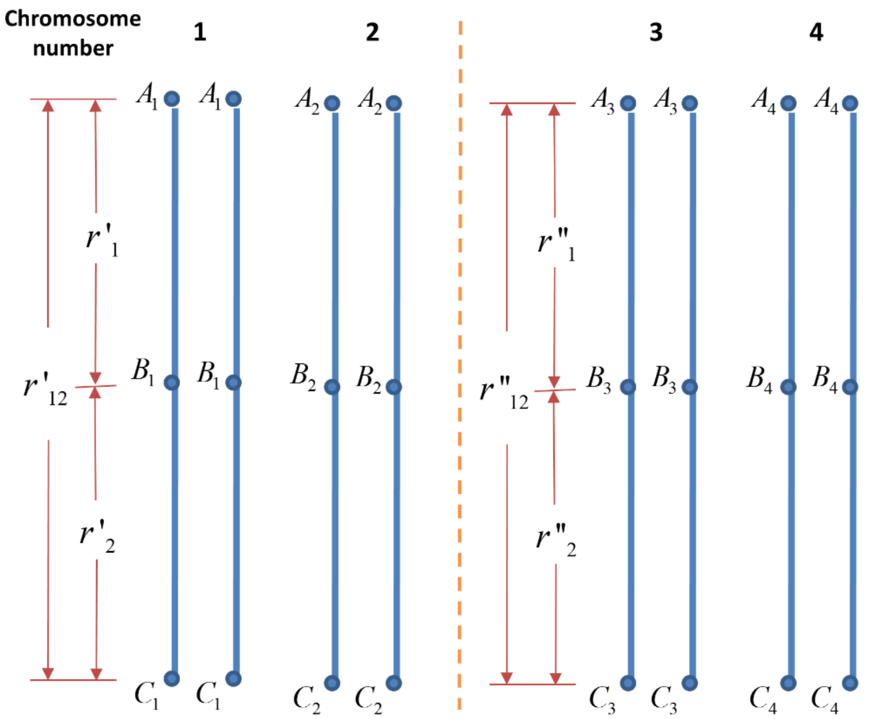

Eight blue lines represent the duplicated homologous chromosomes in an autotetraploid meiosis.

SUPPORTING Note S2. Simulation model and parameters

The method has been described elsewhere to mimic gametogenesis of an autotetraploid individual under bivalent or quadrivalent homologous chromosome pairing in a computer simulation study **(Luo et al. 2001; Luo et al. 2006)**. These programs described in **(Luo et al. 2001; Luo et al. 2006)** are modified to simulate segregation and recombination between genes at any number of loci (markers and QTL) on any number of chromosomes under a given pattern of homologous chromosome pairing (bivalent, quadrivalent or a mixture of the two) in an outbred segregation population of autotetraploid species. Quantitative trait phenotype of an individual in the simulation populations was generated from its simulated genotype at the QTL, genotypic value and a randomly generated number specifying the size of genetic effects at the QTL.

In the present study, we simulated the first generation segregating (S_1) population of 300 individuals from crossing two autotetraploid parental lines. Parental QTL genotypes differed by one ($QQqq \times Qqqq$), two ($QQQq \times Qqqq$), or three ($QQQQ \times Qqqq$) trait increasing alleles denoted as Q. Under the quadrivalent pairing model, the coefficient of double reduction is set equal to zero at the first locus of the simulated linkage map. **Simulation model I** involved a single chromosome of length 100cM carrying 20 bi-allelic markers with allele dosage information known. Parental marker genotypes at each locus are randomly generated, with equal probability for each of the two alleles on each homologous chromosome. The marker data was generated in form with or without allele dosage information as to be specifically informed. These two types of marker data correspond to the case where the marker data is directly collected from genotyping experiments such as GBS (genotyping by sequencing), i.e. marker allele dosage is unknown, or the case where the marker allele dosage is predicted from the genotyping data as illustrated in (Hackett et al 2013). A single QTL is simulated at 34cM (α = 0.15 at the QTL). **Simulation model II** involved a single chromosome of 75 cM length with 10 markers with allele dosage information unknown and a single QTL at 27 cM. In this simulation setting, there were five possible alleles (A-E) and a null allele (O) at each marker locus, each with equal probability. For each set of parents, 200 replicate datasets were generated. The proportion of genetic variance contributed by QTL segregation to the trait phenotype variance in the segregating population will be described case by case.

Supporting Note S3. Variance in the estimation of genetic effect parameters.

Estimated higher level genetic effects have an inherently higher variance compared with estimates of the monogenic effect. Let the mean estimates of the genotypic values for the monogenic, digenic, trigenic and quadrigenic effects in 100 replicate simulated mapping populations be $\hat{G}_0$, …, $\hat{G}_4$ and the corresponding variances be v_0, …, v_4. According to the property of the variance of the sum of independent random variables, the variance of monogenic effect, which is defined as the weighted mean of the differences $G_i^{'} = G_{i+1} - G_i \; (i = 0,...,3)$ with the weights given by $f_i^{'}$ ($\sum_{i=0}^{3} f'_i = 1$), will be equal to $\sum_{i=0}^{3} f'^2_i (v_{i+1} + v_i)$ **(Chen et al 2018)**.

Similarly, the variance of the digenic effect, which is defined as the weighted mean of the differences $G_i^{''} = G_{i+1}^{'} - G_i^{'} = G_{i+2} - 2G_{i+1} + G_i \quad (i = 0,...,2)$ with the weights given by $f_i^{''}$ ($\sum_{i=0}^{2} f''_i = 1$), will be equal to $\sum_{i=0}^{2} f''^2_i (v_{i+2} + 4v_{i+1} + v_i)$. The variance of the trigenic effect, which is defined as the weighted mean of the differences $G_i^{'''} = G_{i+1}^{''} - G_i^{''} = G_{i+2}^{'} - 2G_{i+1}^{'} + G_i^{'} = G_{i+3} - 3G_{i+2} + 3G_{i+1} - G_i \quad (i = 0,1)$ with the weights given by $f_i^{'''}$ ($\sum_{i=0}^{1} f'''_i = 1$), will be equal to $\sum_{i=0}^{1} f'''^2_i (v_{i+3} + 9v_{i+2} + 9v_{i+1} + v_i)$. The variance of the quadrigenic effect, which is defined by $G_0^{''''} = G_1^{'''} - G_0^{'''} = G_4 - 4G_3 + 6G_2 - 4G_1 + G_0$, will be equal to $v_4 + 16v_3 + 36v_2 + 9v_1 + v_0$. It is clear that the variance of higher level genetic effects will be inherently larger than that of lower level genetic effects.

References to Supporting Texts

Chen J, Zhang F, Wang L, Leach LJ, Luo ZW. 2018. Orthogonal contrast based models for quantitative genetic analysis in autotetraploid species. *New Phytol.* **220**: 332-346.

Luo ZW, Hackett CA, Bradshaw JE, McNicol JW, Milbourne D. 2001. Construction of a genetic linkage map in tetraploid species using molecular markers. *Genetics* **157:** 1369-1385.

Luo ZW, Zhang Z, Leach L, Zhang RM, Bradshaw JE, Kearsey MJ. 2006. Constructing genetic linkage maps under a tetrasomic model. *Genetics* **172:** 2635-2645.

Hackett CA *et al.* 2013. Linkage analysis and QTL mapping using SNP dosage data in a tetraploid potato mapping population. *PLoS ONE* **8**, e63939.

SUPPORTING TABLES

Table S1. Probability distribution of diploid gamete genotypes at a QTL and its flanking marker loci from a bivalent meiosis of an autotetraploid individual.

Marker Genotype ($1 \le i \ne j \ne k \ne l \le 4$)	QTL Genotype				Subtotal
	$Q_i\,Q_k$	$Q_i\,Q_l$	$Q_j\,Q_k$	$Q_j\,Q_l$	
$A_iB_i\,/\,A_kB_k$	$\frac{1}{3}\gamma_{00}$	$\frac{1}{3}\gamma_{11}$	$\frac{1}{3}\gamma_{11}$	$\frac{1}{3}\gamma_{22}$	$\frac{1}{3}r_{12}^{'\,2}$
$A_iB_i\,/\,A_kB_l$	$\frac{1}{3}\gamma_{01}$	$\frac{1}{3}\gamma_{10}$	$\frac{1}{3}\gamma_{12}$	$\frac{1}{3}\gamma_{21}$	$\frac{1}{3}r_{12}r_{12}^{'}$
$A_iB_j\,/\,A_kB_k$	$\frac{1}{3}\gamma_{01}$	$\frac{1}{3}\gamma_{12}$	$\frac{1}{3}\gamma_{10}$	$\frac{1}{3}\gamma_{21}$	$\frac{1}{3}r_{12}r_{12}^{'}$
$A_iB_j\,/\,A_kB_l$	$\frac{1}{3}\gamma_{02}$	$\frac{1}{3}\gamma_{11}$	$\frac{1}{3}\gamma_{11}$	$\frac{1}{3}\gamma_{20}$	$\frac{1}{3}r_{12}^{2}$

where $\gamma_{ij} = r_1^i\left(1-r_1\right)^{2-i} r_2^j\left(1-r_2\right)^{2-j}$ ($0 \le i, j \le 2$), r_{12} is the recombination frequency between loci A and B, $r'_{12} = 1 - r_{12}$, and r_1 (or r_2) is the recombination frequency between the QTL and its left (or right) flanking marker.

Table S2. Parameter estimation from BvMethod based on 100 simulations with biallelic SNP markers under a bivalent chromosome pairing model.

Parameter	$QQqq \times Qqqq$		$QQQq \times Qqqq$		$QQQQ \times Qqqq$	
	$h^2 = 0.10$	$h^2 = 0.05$	$h^2 = 0.10$	$h^2 = 0.05$	$h^2 = 0.10$	$h^2 = 0.05$
μ (=10)	9.76 (0.14)	10.20 (0.21)	9.76 (0.14)	9.99 (0.21)	10.03 (0.09)	10.04 (0.13)
θ_1 (=10)	9.23 (0.24)	7.80 (0.54)	9.27 (0.17)	8.84 (0.45)	8.27 (0.25)	5.98 (0.41)
θ_2 (=−6)	-4.95 (0.51)	-5.56 (1.17)	-4.89 (0.66)	-2.65 (0.91)	**0.01 (0.86)**	**0.25 (0.83)**
θ_3 (=3)	1.57 (1.49)	-8.49 (2.80)	**-7.18 (3.15)**	**-11.61 (3.30)**	**-9.05 (2.07)**	**-14.26 (1.64)**
θ_4 (=−1)	--	--	--	--	--	--
$\hat{\sigma}$	24.09 (0.09)	34.46 (0.14)	21.72 (0.09)	31.45 (0.12)	15.04 (0.06)	21.69 (0.08)
σ_{true}	23.90	34.73	21.69	31.51	15.00	21.79
ρ	1.00	1.00	1.00	1.00	1.00	1.00
$Q_{genotype0}$	0.85	0.47	0.71	0.35	0.71	0.20
$Q_{genotype1}$	0.94	0.58	0.99	0.85	0.79	0.37
Accuracy (cM)	0.48 (0.65)	6.79 (1.80)	0.34 (0.61)	4.19 (1.66)	0.34 (0.93)	4.22 (1.85)
Proportion in (± 10 cM)	0.88	0.58	0.87	0.67	0.80	0.60

$Q_{genotype0}$ ($Q_{genotype1}$) represents the proportion of predicted parental QTL genotype configurations exactly matching (or differing by a single allele) the simulated QTL genotypes. The mean and standard errors of parameter estimates are given based on 100 replicate simulations. Values highlighted in bold show parameters that are indeterminable under the correct parental QTL genotype configuration, but have been estimated due to incorrect prediction of QTL genotype configuration. ρ is the empirical power. Accuracy (cM) is the distance between the true QTL location and its inferred location. -- denotes indeterminable parameters.

Table S3. Parameter estimation from QvMethod based on 100 simulations with biallelic SNP markers under a quadrivalent chromosome pairing model.

Parameters	$QQqq \times Qqqq$		$QQQq \times Qqqq$		$QQQQ \times Qqqq$	
	$h^2 = 0.10$	$h^2 = 0.05$	$h^2 = 0.10$	$h^2 = 0.05$	$h^2 = 0.10$	$h^2 = 0.05$
μ (=10)	9.87 (0.17)	9.81 (0.21)	10.05 (0.14)	9.79 (0.18)	9.90 (0.11)	9.99 (0.16)
θ_1 (=10)	9.10 (0.29)	7.90 (0.59)	9.45 (0.22)	8.22 (0.50)	7.97 (0.26)	6.68 (0.44)
θ_2 (=−6)	-6.36 (0.48)	-3.35 (0.88)	-5.71 (0.51)	-5.41 (0.84)	-2.33 (0.72)	-1.72 (0.81)
θ_3 (=3)	2.53 (0.91)	-3.65 (2.09)	0.97 (1.12)	-0.86 (1.87)	**-5.95 (1.34)**	**-4.68 (1.59)**
θ_4 (=−1)	-2.48 (4.15)	17.26 (8.11)	-2.68 (3.31)	-2.11 (6.47)	**-9.65 (5.16)**	**-8.10 (6.03)**
$\hat{\sigma}$	27.55 (0.11)	39.66 (0.14)	25.36 (0.10)	36.49 (0.18)	17.24 (0.07)	24.99 (0.09)
σ_{true}	27.63	40.15	25.44	36.96	17.37	25.24
ρ	1.00	1.00	1.00	1.00	1.00	1.00
$Q_{genotype0}$	0.85	0.47	0.72	0.36	0.60	0.24
$Q_{genotype1}$	0.94	0.57	0.98	0.79	0.80	0.49
Accuracy (cM)	-1.56 (0.90)	7.38 (1.86)	1.37 (0.90)	6.15 (1.66)	0.17 (1.01)	6.41 (1.95)
Proportion in (± 10 cM)	0.84	0.44	0.85	0.60	0.78	0.54

$Q_{genotype0}$ ($Q_{genotype1}$) represents the proportion of predicted parental QTL genotype configurations exactly matching (differing by a single allele) the simulated QTL genotypes. The mean and standard errors of parameter estimates are given based on 100 replicate simulations. Values highlighted in bold show parameters that are indeterminable under the correct parental QTL genotype configuration, but have been estimated due to incorrect prediction of QTL genotype configuration. ρ is the empirical power. Accuracy (cM) is the distance between the true QTL location and its inferred location.

Table S4. Parameter estimation from BvMethod based on 100 simulation with multi-allelic markers under a bivalent chromosome pairing model.

Parameter	$QQqq \times Qqqq$		$QQQq \times Qqqq$		$QQQQ \times Qqqq$	
	$h^2 = 0.10$	$h^2 = 0.05$	$h^2 = 0.10$	$h^2 = 0.05$	$h^2 = 0.10$	$h^2 = 0.05$
$\mu = 10$	10.07 (0.14)	10.08 (0.22)	10.01 (0.13)	9.76 (0.19)	9.96 (0.09)	10.08 (0.13)
$\theta_1 = 10$	9.62 (0.24)	7.91 (0.59)	9.61 (0.20)	8.66 (0.41)	8.37 (0.38)	6.68 (0.42)
$\theta_2 = -6$	-5.56 (0.48)	-5.29 (1.07)	-5.74 (0.63)	-4.16 (0.97)	**0.58 (0.77)**	**-0.61 (0.73)**
$\theta_3 = 3$	3.28 (1.51)	-7.30 (2.96)	**-10.02 (3.53)**	**-10.29 (2.81)**	**-11.12 (2.54)**	**-13.14 (2.23)**
$\theta_4 = -1$	--	--	--	--	--	--
$\hat{\sigma}$	23.88 (0.11)	34.11 (0.15)	21.59 (0.09)	31.16 (0.13)	14.92 (0.06)	21.53 (0.10)
σ_{true}	23.90	34.75	21.69	31.51	15.00	21.79
ρ	1.00	1.00	1.00	1.00	1.00	1.00
$Q_{genotype0}$	0.90	0.53	0.74	0.26	0.65	0.22
$Q_{genotype1}$	0.97	0.60	0.99	0.82	0.71	0.35
Accuracy (cM)	0.95 (0.82)	5.80 (1.53)	0.56 (0.68)	6.05 (1.75)	1.47 (0.86)	4.58 (1.63)
Proportion in ($\pm$ 10 cM)	0.83	0.62	0.86	0.61	0.84	0.61

$Q_{genotype0}$ ($Q_{genotype1}$) represents the proportion of predicted parental QTL genotype configurations exactly matching (differing from by only a single allele) the simulated QTL genotype. The mean and standard errors of parameter estimates are given based on 100 replicate simulations. Values highlighted in bold show the parameters that are indeterminable under the correct parental QTL genotype configuration, but have been estimated due to incorrect prediction of QTL genotype configuration. ρ is the empirical power. Accuracy (cM) is the distance between the true QTL location and its inferred location. -- denotes indeterminable parameters.

Table S5. Parameter estimation from QvMethod based on 100 simulations with multi-allelic markers under a quadrivalent chromosome pairing model.

Parameter	$QQqq \times Qqqq$		$QQQq \times Qqqq$		$QQQQ \times Qqqq$	
	$h^2 = 0.10$	$h^2 = 0.05$	$h^2 = 0.10$	$h^2 = 0.05$	$h^2 = 0.10$	$h^2 = 0.05$
$\mu = 10$	10.15 (0.16)	9.82 (0.25)	10.20 (0.16)	10.41 (0.23)	10.10 (0.11)	9.85 (0.16)
$\theta_1 = 10$	9.62 (0.30)	8.34 (0.57)	9.48 (0.27)	8.16 (0.52)	8.31 (0.43)	7.38 (0.41)
$\theta_2 = -6$	-5.70 (0.56)	-2.70 (1.20)	-4.57 (0.45)	-3.22 (1.14)	-4.39 (0.77)	0.36 (0.95)
$\theta_3 = 3$	-2.37 (1.43)	-9.53 (2.55)	-1.32 (1.01)	-8.35 (2.01)	**-5.53 (1.81)**	**-7.55 (1.80)**
$\theta_4 = -1$	2.38 (4.05)	-6.93 (7.99)	6.36 (3.32)	3.50 (7.26)	**-2.52 (5.47)**	**-6.21 (5.58)**
$\hat{\sigma}$	27.39 (0.10)	39.73 (0.15)	25.37 (0.10)	36.53 (0.15)	17.33 (0.07)	24.68 (0.10)
σ_{true}	27.63	40.15	25.44	36.96	17.37	25.24
ρ	1.00	1.00	1.00	1.00	1.00	1.00
$Q_{genotype0}$	0.77	0.41	0.76	0.25	0.56	0.22
$Q_{genotype1}$	0.91	0.56	0.98	0.76	0.71	0.55
Accuracy (cM)	1.38 (1.06)	7.30 (1.67)	2.75 (1.02)	6.51 (1.64)	3.65 (1.21)	6.97 (1.40)
Proportion in ($\pm$ 10 cM)	0.75	0.49	0.76	0.54	0.70	0.59

$Q_{genotype0}$ ($Q_{genotype1}$) represents the proportion of predicted parental QTL genotype configurations exactly matching (differing from by only a single allele) the simulated QTL genotype. The mean and standard errors of parameter estimates are given based on 100 replicate simulations. Values highlighted in bold are the parameters that are indeterminable under the correct parental QTL genotype configuration, but have been estimated due to incorrect prediction of QTL genotype configuration. ρ is the empirical power. Accuracy (cM) is the distance between the true QTL location and its inferred location.

Table S6. Parameter estimation from BvMethod or QvMethod based on 200 repeated simulations under a mixed chromosome pairing model with simulated parental QTL genotypes $QQQq \times Qqqq$ **.**

Parameter (=true value)	$\lambda = 0.00$		$\lambda = 0.25$		$\lambda = 0.50$		$\lambda = 0.75$		$\lambda = 1.00$	
	BvMethod	QvMethod	BvMethod	QvMethod	BvMethod	QvMethod	BvMethod	QvMethod	BvMethod	QvMethod
μ (=10)	10.12 (0.10)	10.14 (0.10)	9.75 (0.10)	9.56 (0.10)	9.75 (0.10)	9.78 (0.13)	9.87 (0.11)	9.39 (0.19)	9.79 (0.11)	10.03 (0.11)
θ_1 (=10)	8.76 (0.17)	8.55 (0.16)	8.03 (0.18)	8.90 (0.17)	8.02 (0.22)	9.06 (0.17)	8.27 (0.22)	9.25 (0.18)	7.41 (0.23)	9.37 (0.15)
θ_2 (=−6)	-4.58 (0.40)	-4.78 (0.40)	-4.29 (0.40)	-4.95 (0.44)	-3.60 (0.47)	-5.04 (0.38)	-3.66 (0.47)	-5.13 (0.35)	-2.83 (0.48)	-5.43 (0.30)
θ_3 (=3)	-6.99 (1.79)	-6.15 (1.84)	-5.38 (1.44)	-0.62 (1.01)	-7.25 (1.99)	1.53 (0.80)	-7.58 (1.88)	2.09 (0.76)	-10.84 (1.36)	1.86 (0.74)
θ_4 (=−1)	--	--	--	-1.18 (2.77)	--	-3.62 (2.24)	--	0.87 (2.46)	--	0.38 (2.25)
σ	21.62 (0.07)	21.64 (0.07)	22.83 (0.07)	22.61 (0.07)	23.99 (0.07)	23.63 (0.07)	25.11 (0.07)	24.69 (0.07)	25.75 (0.08)	25.25 (0.08)
σ_{true}	21.69		22.67		23.60		24.50		25.35	
Incompatible markers	--	--	0.0070 (0.0002)	--	0.0140 (0.0002)	--	0.0214 (0.0003)	--	0.0285 (0.0003)	--
ρ	1.00	1.00	1.00	1.00	1.00	1.00	0.99	1.00	0.98	1.00
$Q_{genotype0}$	0.660	0.665	0.495	0.680	0.445	0.675	0.440	0.715	0.290	0.755
$Q_{genotype1}$	0.980	0.980	0.965	0.990	0.915	0.975	0.890	0.980	0.865	0.995
Accuracy (cM)	1.66 (0.66)	1.75 (0.73)	2.90 (0.62)	2.75 (0.54)	4.73 (0.85)	2.17 (0.59)	4.94 (0.90)	3.05 (0.65)	5.19 (1.03)	2.56 (0.66)
Proportion in (±10 cM)	0.845	0.830	0.840	0.860	0.755	0.840	0.750	0.820	0.645	0.855

The proportion of quadrivalent chromosome pairing is given by λ. $Q_{genotype0}$ ($Q_{genotype1}$) represents the proportion of predicted parental QTL genotype configurations exactly matching (or differing by only a single allele) the simulated QTL genotypes. The mean and standard errors of parameter estimates are given based on 200 replicate simulations. Individual marker genotypes that are unexpected from the theoretical genotype distribution expected under a model of bivalent chromosome pairing are removed from the simulated datasets before analysis with BvMethod. ρ is the empirical power. Accuracy (cM) is the distance between the true QTL location and its inferred location. Heritability was equal to 10%.

Table S7. Parameter estimation from BvMethod or QvMethod based on 200 repeated simulations under a mixed chromosome pairing model with simulated parental QTL genotypes $QQqq \times qqqq$.

Parameter	$\lambda = 0.00$		$\lambda = 0.25$		$\lambda = 0.50$		$\lambda = 0.75$		$\lambda = 1.00$	
(=true value)	BvMethod	QvMethod	BvMethod	QvMethod	BvMethod	QvMethod	BvMethod	QvMethod	BvMethod	QvMethod
μ (=10)	9.94 (0.07)	9.95 (0.11)	9.92 (0.07)	9.84 (0.07)	10.00 (0.08)	9.96 (0.08)	9.85 (0.08)	9.83 (0.08)	10.03 (0.09)	9.99 (0.09)
θ_1 (=10)	8.57 (0.24)	8.31 (0.25)	8.19 (0.22)	8.05 (0.25)	7.49 (0.26)	7.89 (0.24)	7.49 (0.27)	8.01 (0.23)	7.62 (0.24)	8.12 (0.26)
θ_2 (=−6)	-4.98 (0.31)	-4.88 (0.32)	-4.09 (0.34)	-4.49 (0.39)	-3.36 (0.39)	-3.80 (0.39)	-4.02 (0.40)	-4.86 (0.46)	-3.75 (0.42)	-4.13 (0.36)
θ_3 (=3)	**2.67 (2.60)**	**3.97 (2.17)**	**0.93 (1.65)**	**2.15 (1.12)**	**2.12 (1.99)**	**-0.82 (1.28)**	**0.37 (1.68)**	**0.62 (1.14)**	**1.58 (1.36)**	**0.97 (1.17)**
θ_4 (=−1)	--	**--**	--	**-4.77 (4.06)**	--	**-1.86 (4.46)**	--	**-3.64 (4.13)**	--	**0.34 (3.79)**
σ	17.96 (0.06)	18.01 (0.08)	18.51 (0.06)	18.44 (0.06)	19.21 (0.06)	19.09 (0.06)	19.89 (0.06)	19.77 (0.07)	20.43 (0.06)	20.22 (0.06)
σ_{true}	17.83		18.46		19.07		19.65		20.21	
Incompatible markers	--	--	0.0072 (0.0002)	--	0.0143 (0.0002)	--	0.0216 (0.0003)	--	0.0288 (0.0003)	--
ρ	1.00	1.00	1.00	1.00	1.00	1.00	1.00	1.00	0.99	1.00
$Q_{genotype0}$	0.775	0.740	0.645	0.635	0.620	0.620	0.550	0.620	0.555	0.620
$Q_{genotype1}$	0.930	0.940	0.930	0.940	0.870	0.910	0.850	0.875	0.895	0.915
Accuracy (cM)	0.57 (0.60)	0.36 (0.69)	0.64 (0.83)	0.70 (0.75)	-2.63 (0.89)	1.69 (0.80)	-4.16 (0.92)	1.50 (0.83)	-5.44 (0.94)	2.64 (0.96)
Proportion in (±10 cM)	0.860	0.835	0.710	0.830	0.540	0.745	0.445	0.770	0.360	0.760

The proportion of quadrivalent chromosome pairing is given by λ . $Q_{genotype0}$ ($Q_{genotype1}$) represents the proportion of predicted parental QTL genotype configurations exactly matching (or differing by a single allele) the simulated QTL genotypes. The mean and standard errors of parameter estimates are given based on 100 replicate simulations. Individual marker genotypes that are unexpected from the theoretical genotype distribution expected under a model of bivalent chromosome pairing are removed from the simulated datasets before analysis with BvMethod. ρ is the empirical power. Accuracy (cM) is the distance between the true QTL location and its inferred location. Heritability was equal to 10%.
